# Ambient temperature as a factor contributing to the developmental divergence in sympatric salmonids

**DOI:** 10.1371/journal.pone.0258536

**Published:** 2021-10-15

**Authors:** Evgeny V. Esin, Grigorii N. Markevich, Nikolai O. Melnik, Dmitriy V. Zlenko, Fedor N. Shkil

**Affiliations:** 1 A.N. Severtsov Institute of Ecology and Evolution RAS, Moscow, Russian Federation; 2 Kronotsky Nature Biosphere Reserve, Yelizovo, Kamchatka region, Russian Federation; 3 Lomonosov Moscow State University, Moscow, Russian Federation; 4 Koltzov Institute of Developmental Biology RAS, Moscow, Russian Federation; University of Iceland, ICELAND

## Abstract

Factors and mechanisms promoting resource-based radiation in animals still represent a main challenge to evolutionary biology. The modifications of phenotype tied with adaptive diversification may result from an environmentally related shift having occurred at the early stage of development. Here, we study the role of temperature dynamics on the reproductive sites in the early-life divergence and adaptive radiation of the salmonid fish *Salvelinus malma* dwelling in the Lake Kronotskoe basin (North-East Asia). Local sympatric charr ecomorphs demonstrate strict homing behaviour guiding the preordained distribution along tributaries and, hence, further development under different temperatures. We thoroughly assessed the annual temperature dynamics at the spawning grounds of each morph as compared to an ancestral anadromous morph. Then we carried out an experimental rearing of both under naturally diverging and uniformed temperatures. To compare the morphs’ development under the dynamically changing temperatures, we have designed a method based on calculating the accumulated heat by the Arrhenius equation. The proposed equation shows a strong predictive power and, at the same time, is not bias-susceptible when the developmental temperature approximates 0°C. The temperature was found to significantly affect the charrs’ early ontogeny, which underlies the divergence of developmental and growth rates between the morphs, as well as morph-specific ontogenetic adaptations to the spawning site’s temperatures. As opposed to the endemic morphs from Lake Kronotskoe, the anadromous *S*. *malma*, being unexposed to selection оn highly specific reproduction conditions, showed a wide temperature tolerance, Our findings demonstrate that the hatch, onset timing of external feeding, and size dissimilarities between the sympatric morphs reveal themselves during the development under contrast temperatures. As a result of the observed developmental disparities, the morphs occupy specific definitive foraging niches in the lake.

## Introduction

Pinning down the factors and mechanisms promoting phenotypic divergence in animals have represented a challenge for evolutionary biology. The origin of adaptive polymorphism is rooted in the interplay between the environmental factors and genetically bound developmental regulators [1–4]. Even minor shifts in the early developmental rate and timing can promote a definitive phenotype modification allowing for evolutionary radiation [2,3,5,6]. Thus, adaptive deviations of ontogeny have gained particular attention [7,8]. An abundant comparative material on the ontogeny deviations is represented by the fishes invaded into the newly formed lacustrine ecosystems [9]. Although research on the factors underpinning the origin of the ecologically and morphologically different species/morphs of lacustrine fishes has been extensive and fruitful recently, many environmental predictions regarding the drivers of adaptive radiation still remain unverified [10–15].

In the present paper, we explore the effect developmental temperatures produce on diversification of *Salvelinus malma* charr (Salmonidae) dwelling in the Lake Kronotskoe (LK) basin in North-East Asia. The local charrs demonstrate a remarkable diversity achieved through multiple diversification modes typical of the Northern Hemisphere fishes: pelagic-benthic, shallow-deep water, and lacustrine-riverine. Thus, the species seems to be one of the brightest examples of adaptive radiation in the high-latitude ecosystems [16]. The lake has been inaccessible to invasive fishes for twelve thousand years due to huge rapids in the outflowing river [17], and intact since the Kronotsky state reserve was established in 1934. The LK charrs are currently represented by eight true-breeding morphs, five of which spawn at the remote lake tributaries and migrate to the lake in youth for foraging and maturation [18]. The ancestral morph of *S*. *malma* - Dolly Varden (DV) spawns at the mountain river sections of the surrounding open-water basins and migrates to the sea for maturation [19]. Both ancestral DV and the LK charrs exhibit a strict reproductive philopatry and return to their parents’ spawning sites [20]. The preordained distribution of breeding fish along the hydrologically heterogeneous tributaries results in the morphs’ early development under various environmental conditions, among which temperature dynamics seems to be most crucial.

The ambient temperature strongly affects the level of metabolic activity and oxygen consumption of poikilothermic animals [21–25]. It regulates such vital biological processes as transcription and chromatin organization [26–29], catalytic activity and enzyme folding [30–32]. Hence, it largely orchestrates the process of adaptive specialization in the heterogeneous environment. In sympatric fishes, the ambient temperature differences commonly cause dissimilarities in the developmental timing and growth [33–36].

Considering the pertinent literature, we hypothesize that the difference in the early-development temperatures of the LK charrs promotes their phenotypic divergence. Temperature affects the morphs’ growth and development rates enhancing their differences in size and morphological advancement. This may predetermine the dissimilarity in fitness condition and allows to occupy different ecological niches in the lake. To test this assumption, we measured the temperature dynamics at the charrs’ reproduction sites and confirmed its specifics for each morph (including DV) (i). Then we performed a series of experiments to evaluate the influence of the ambient temperature on the developmental timing and growth rate. Firstly, to evaluate the range of ancestral developmental variability, we reared DV under its natural temperature regime and the regimes typical of the LK morphs’ reproduction sites (ii). Then, we reared the presumably more specialized LK morphs under their natural temperature regimes (iii). To evaluate the inherited capacity to adapt to specific developmental temperatures, we additionally reared DV and the LK morphs at the same standard temperature (iv), being the lower limit of the optimal temperature range reported for *S*. *malma* [37–39]. In all the experimental series run, we assessed and compared both developmental and somatic growth rates.

## Materials and methods

### Ecosystem description

Lake Kronotskoe (N 54.822; E 160.246) is a big (surface area 246 km^2^) and deep (up to 136 m) landlocked waterbody. The outflowing river passes through huge rapids with a total drop of more than 100 m [40]. The whole LK catchment area (2 330 km^2^) has a very stable hydrological regime maintained by the local climate depending on the breeze circulation [41,42]. The lake tributaries never freeze solid in winter; their thermal fluctuations are stabilized by groundwater discharges determined by the old lava flows’ arrangement [41]. Judging by our previous observations, we may suppose that the lake tributaries are regularly flooded at the end of May–beginning of July (see supplementary **S1 Fig** for further details).

**Fig 1:**
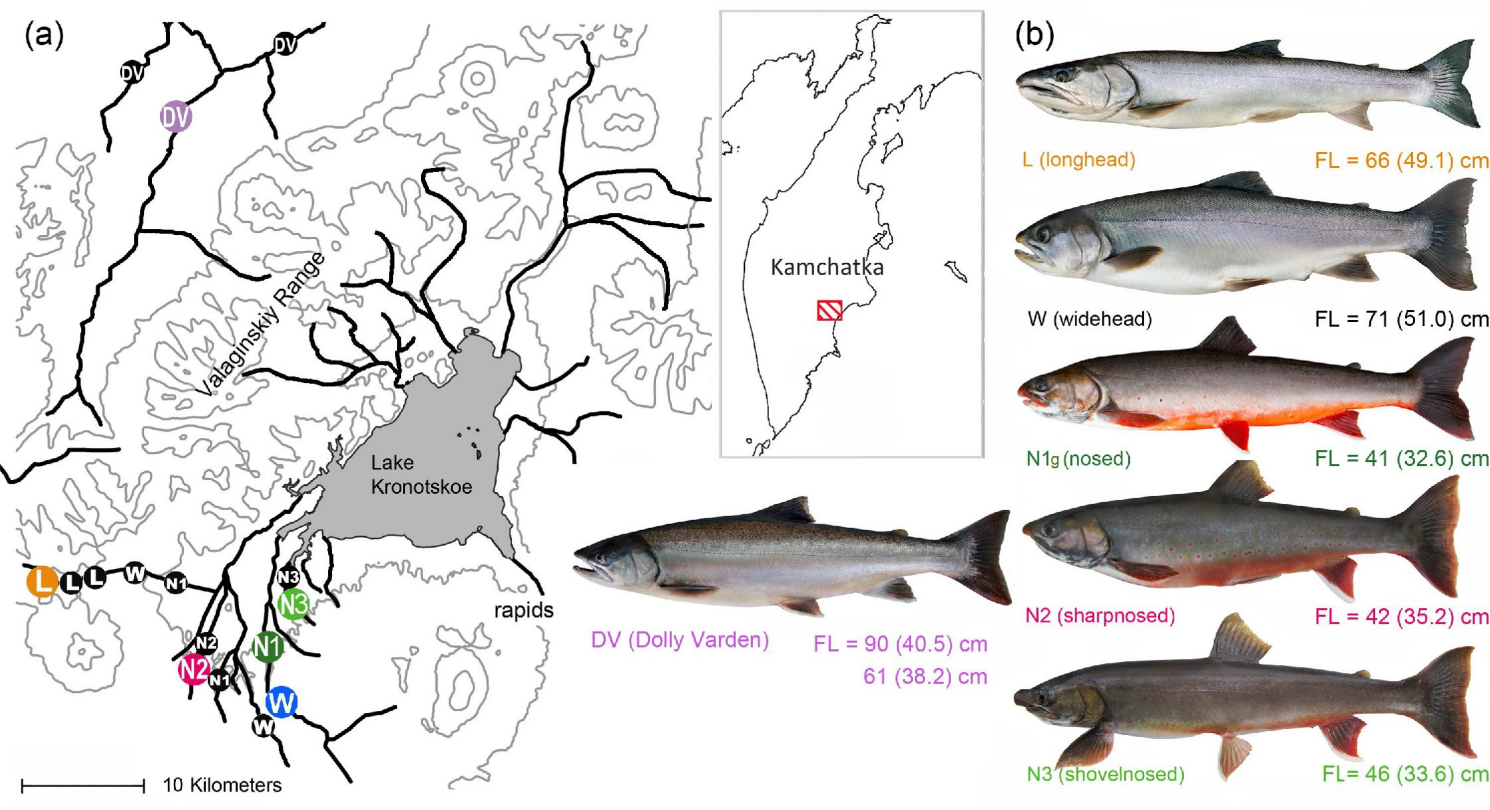
The area under investigation and the location of the reproduction sites, where the temperature dynamics was analyzed (**a**; positions of the spawning grounds for fish sampling are put in colour); the adult fish external appearance (**b**; maximal (mean) fork length is shown for the morphs, Dolly Varden’s fork length–for the river where the spawners were caught (Kamchatka River, above), and for the Kronotskaya River population (below)).

The LK fish fauna consists of native endemic kokanee, *Oncorhynchus nerka*, and charrs [43]. Among the latter, the obligatory piscivorous “longhead” morph (L) spawns in the headwaters. The facultative piscivorous “widehead” charr (W, also “white”) reproduces in the upper course of the tributaries. Three “nosed” benthivorous morphs (N1g, N2, N3) prefer to spawn in the middle course: N1g morph uses various tributaries all over the basin, while N2 and N3 morphs occupy discrete reproduction sites in two remote spring brooks (**Fig 1**) [18,44]. The morphs show strict homing and timing of reproduction; the exact localization of reproduction sites was revealed through visual observations and samplings performed in 2011–2019 [20]. As a valid criterion of a spawning site, we used the annual presence of dozens of breeding individuals (no less than 20-30); a complete absence of spawning pairs signified the spawning sites’ borders. The spawning aggregation of different morphs was never observed [20]. During the nine-year period, the spawning peak occurred in the middle of September (L, N1g, N3) or a week later (W and DV), and only N2 morph spawned at the end of August (**S2 Fig**). All the morphs had a long-lasting early ontogenic period in the spawning nests (i.e. redds), and left them, settling on the nearest sites, the next year after fertilization [45].

### Field material collection

The charr *Salvelinus malma* is not an endangered or protected species in the Russian Federation. Following the Federal law “On Fisheries and Conservation of Aquatic Biological Resources” №166-ФЗ, the non-commercial fishing of this charr does not require any permissions. All field and experimental procedures with fish were carried out according to the guidelines and following the laws and ethics of the Russian Federation, and approved by the ethics committee of the Severtsov Institute of Ecology and Evolution, Russian Academy of Sciences.

Spawners were caught using scoop-nets on the previously allocated spawning grounds [20]. DV spawning site was chosen in the nearest watercourse draining opposing the slope of the Valaginskiy range (**Fig 1**). We monitored the breeding dynamics by assessing the spawners’ density and collected fish at the peak of spawning. The artificial breeding was performed by mixing sexual products obtained from three middle-sized females and 3-5 males using the dry method [46]. In all cases, the fertilization rate was about 90%. To reduce the family variance effect, we mixed the fertilized eggs of different females per morph. Then, in 48 h, the eggs were placed in thermostatic containers (~3⁰C; no less than 400 of each morph) and delivered to the laboratory.

### Developmental temperatures

The temperature in the redds was measured hourly during the annual period using Starmon mini temperature loggers (Star Oddi, Iceland) with an absolute accuracy at least 50 mK according to the reference XRX 620 device (RBR, Canada). The loggers were placed inside the perforated steel pipes hammered into the redds to the depth of 15 cm. We set sensors (one logger per site) at three separate spawning sites of DV, W, L and N1g morphs, and in two maximally remote redds at the N2 and N3 morphs spawning sites (**Fig 1**). Then, the data from each logger was averaged daily to obtain the annual temperature dynamics at each redd in order to compare mean temperatures at different sites (**S3 Fig**).

To confirm the validity of averaged temperatures obtained from different loggers for the morphs, we analyzed the rate of temperature change (slope) in each redd. To do so, we assessed the time course of the first derivative of the daily water temperature in four periods: in winter (November 15 to February 15), spring (May 15 to June 14), summer (June 15 to July 31) and autumn (September 15 to October 31). The data were processed in Python surrounding using numpy and visualized in Matplotlib. After that, we calculated the mean redd-specific rate of temperature change for each period and classified the resultant values of spawning sites. The cluster analysis (Euclidean metrics) was performed in StatSoft v.10 [47]. Additionally, to understand whether the temperature variation was more between than within the morphs’ reproduction sites, we compared the standard deviation of daily-averaged values observed at the spawning sites of distinct morphs with the parameters calculated for all the spawning sites in our study.

### Experimental design

**Fig 2:**
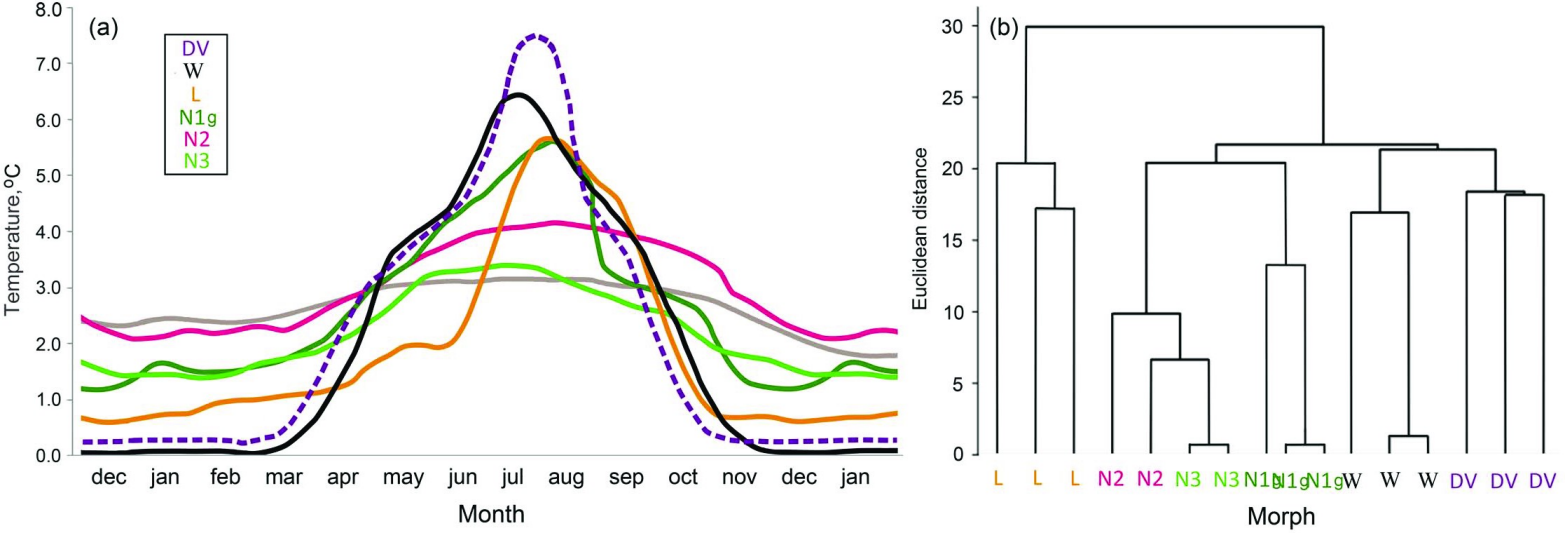
Annual dynamics of mean daily temperatures at the bottom water level at the spawning grounds of the Lake Kronotskoe charr morphs and Dolly Varden (**a**; coloured for different morphs, the standard temperature regime of rearing is shown in grey); clustering mean velocity of temperature change in winter, spring, summer and autumn to differentiate the spawning sited of the Lake Kronotskoe charr morphs and the anadromous Dolly Varden (**b**).

In the first experiment (duration 45 weeks), we reared DV under the natural temperature regime, as well as under the temperature regimes specific to each LK morph (**Fig 2A**). The control unit (ERG, Russia) with a relative sensitivity of 2 mK, duplicated for reliability, provided a temperature control. The hatchery maintained the controlled temperature with an accuracy of ±0.03°C in each tank (see **S4 Fig** for further details). The in-house developed software monitored the dynamics of water tanks cooling and the amount of heat required to maintain the temperature (which was performed by a setup comprising 120 W heating elements). Constant temperature to be maintained was -3 - +2°C by industrial air-cool system SM232 (Polair, Russia).

In the second experiment (60 weeks), we reared five LK morphs under the natural temperature regimes specific to the morph’s spawning sites.

In the third experiment (40 weeks), DV and LK morphs were reared at a standard temperature of ~2.9°C (**Fig 2A**, grey line, = ST regime), which is the lower limit of the *S*. *malma* optimal temperature range [37–39] and still higher than the temperatures registered at the LK spawning sites in winter-spring. A couple of the external chillers (Hailea HC 500A) controlled this temperature regime with an accuracy of ±0.3°C.

All other rearing conditions in the experimental series were the same. We used 250-l water tanks filled with the UV-treated soft water (150 ppm, pH = 7.8, oxygen ≥ 11 mg l^-1^) with the filtration intensity of 900 l h^-1^. Eggs of each morph were placed in three trays to ensure the experiments’ replication. The newly hatched fish were transferred into mesh cages with the stocking density of 100 individuals per 0.004 m^3^ (25 000 _*_ m^-3^). Following Johnston [48], the fish were resettled with a twice-smaller density at 15 weeks after hatching. Embryos were reared in the dark. The lighting regime was changed to 10 h day: 14 h night (Sun-Glo lamps, Hagen) simultaneously with the start of feeding. All fish were fed with standard weighted portions of *Artemia salina* nauplii in the first two weeks and then with chironomid larvae.

The mortality rate was rather consistent in all the experimental series: ~25% to the moment of hatching and ~10% during the transition to the external feeding. Therefore, the mortality factor was neglected while assessing the morphs’ development.

### Developmental staging

Following Balon [49] in modifications, we identified two prenatal and six early postnatal developmental stages typical of salmonids: early embryo (1), formed embryo after eye pigmentation (2), hatched free embryo (3), late free embryo (4), feeding alevin (5), late alevin (6), fry (7, = juvenile) and late fry (8, = ‘parr’) (see **S5 Fig** and **S1 Table** for detailed stage description). In addition, we defined an alleged timing of leaving the redd, which is characterized by 50% depletion of the yolk sack in the absence of feeding [45].

### Developmental rate evaluation

The rate of stage transition in fish is the parameter influenced by temperature [50,51]. At a constant temperature, the development could be considered as a linear function of time, i.e. in terms of the degree-days (dd) accumulation [52]. We assessed the dd values for each experimental series at the moment when 50% of fish reached the next developmental stage. Meanwhile, this approach leads to a high bias if the developmental temperature varies and approximates to 0°C [53]. In our experiments, DV accumulated 380 dd until hatching when incubated at ~2.9°C, and only 110 dd when incubated at ~0.5°C. Having occurred in all morphs, this bias clearly indicated the failure of the classical approach in case of varying temperature (**S6 Fig**).

The relationship between the temperature and developmental rate could be levelled by equations, such as parabolic, power, and exponential ones [50,51,53–55]. Therefore, at varying temperatures the dd scale cannot ensure properly comparing the rate of charrs development, which is generally determined by the rate of biochemical processes. The temperature dependence of the developmental rate should be in accordance with the Van ‘t Hoff rule. The rate and temperature of biochemical reactions are related by the Arrhenius equation *k* = *Ae* ^-*Ea*/*RT*^, where T is the absolute temperature (K), A–some dimension factor, Ea–activation energy, and R–universal constant, = 8.31 J mol^-1^ K^-1^. This approach to assess the dependency between the development rate and the temperature was successfully used for approximation of the experimental data on the salmonids development [54,55]. Associating the development with the accumulation of some chemical reaction product [56], the amount of this product (D) would describe the “degree of development” of the organism at a given time (τ):

$$D(\tau )=\int_{0}^{\tau }A\cdot e^{\frac{-E_{a}}{R\cdot T(t)}}dt$$

To apply the aforementioned approach the dimension factor ‘A’ and activation energy ‘Ea’ are to be defined. For this purpose, we used the developmental data obtained during DV rearing under its specific temperature regime, regimes typical of each LK morphs, and ST regime (**Fig 2**). We defined six crucial developmental points: 50% eye pigmentation, hatching, swimming onset, foraging onset, late alevins absorb sacks and transition to the fry stage. Assuming D = 1.0 at the peak of hatching, we applied a fitting procedure to determine A and Ea considering all five independent datasets. It was performed by the error function calculated as the sum of differences between actual D at the given A and Ea, and some iteratively defined D values corresponding to each developmental point. This algorithm minimized the error function selecting the unknown A and Ea, and equaled D to the control DV values. After that, fitting was implemented in Python surrounding using numpy and scipy libraries for the hatching, alevin, fry, hatching + late embryo, hatching + alevin, and hatching + alevin + fry stages.

The obtained values of A and Ea were used to estimate D(τ) in the LK morphs’ experimental series at the moment when 50% of fish reached the next developmental stage. Hereafter in this work, the terms “degree of development” or simply “development” refer to the corresponding value of D–a quantitatively and indiscretely generalized Arrhenius-based assessment of “how far the fish development has gone”.

### Growth rate evaluation

In all experimental series, fish were randomly sampled at the moment when 50% of them had reached the next developmental stage, and additionally at two developmental points fixed at D(τ) value (**S2 Table**). Each sample included 15 hungry fish euthanized by lidocaine (cas 137-58-6, Merch) and photographed. To measure the fork length (the length from the snout tip to the caudal fin notch, FL, ±0.1 mm), we used ImageJ v.1.52 [57]. The weight (W) was measured with balance HR-AZ (AND) with an accuracy of ±0.001 g. In total, 11940 individuals were analyzed. Hereafter in this work, the terms “growth” or “growth rate” refer only to the physical length and/or weight of the fish, but not to its developmental stage.

To compare FL and W of the morphs, we used ANOVA and multiple post-hoc Tukey HSD or paired t-test in StatSoft v.10. To assess and calculate the effect of temperature on growth and development, we evaluated the Spearman correlation between the accumulated heat (dd) and FL/D(τ) increments using the developmental segments from **S3 Table** for the experimental series combined.

## Results

### Natural temperatures of early development

The loggers’ data demonstrated drastic dissimilarities between the temperature dynamics at the spawning sites belonging to different morphs. The DV’s spawning sites were characterized by cooling to the average of 0.8°C in winter and heating up to 7.5°C in summer. The range of temperature variability of piscivorous morphs’ spawning grounds resembled those at DV sites. However, extremely low winter temperatures (0.1°C on average) were registered at the W morph’s spawning grounds. A significant (about one month) prolongation of the winter period (~0.4°C) along with very cold conditions of the flood (**S3 Table**) were observed at the L morph’s sites. The annual temperature fluctuations at the benthivorous nosed morphs’ reproduction sites (N1g and especially N2 and N3) appeared to be much smaller (1.3-5.7°C). The N2 morph’s spawning sites demonstrated the warmest temperatures during wintertime, until the end of April (~2.3°C in contrast to ~1.5-1.6°C typical for N1g and N3). Since April, the warmest conditions were recorded for the W morph’s and DV’s reproductive sites (**S3 Table**). Overall, the temperature variability between spawning sites belonging to a distinct morph was significantly lower than the one between all spawning sites under study (for more details see **S7 Fig**).

The analysis of temperature change rate (slope) (**S8 Fig**) revealed that DV, W and L morphs were largely reproducing at the sites with an intensive seasonal warming and cooling, while the nosed morphs were found at the sites with a reduced rate of warming and cooling. The results of cluster analysis showed that the temperature dynamics at spawning sites of different morphs tended to exceed significantly the one at the spawning grounds exploited by each of the six morph solely (**Fig 2B**). This finding allowed us to smooth the data obtained from loggers placed at the spawning sites exploited by one morph with a week-time step, to suppress occasional temperature fluctuations and refer to these temperatures in artificial rearing of LK morphs and DV (**Fig 2A**).

### Dolly Varden developmental variability under different temperature regimes

#### Developmental rate

The timing of DV development depended on the temperature regime (**S4 Table**): hatching took place from 113^rd^ to 189^th^ day after fertilization (ST regime and W regime correspondingly); external feeding onset occurred from 159^th^ (ST regime) to 241^st^ day (W regime); and transition to the fry stage–from 240^th^ (ST regime) to 314^th^ day (L regime). The difference in dd values between the DV series reared under different temperatures showed further increase. By the time of hatching, cold-water series (DV, W and L regimes) on average gained 35% of the dd value obtained by warm-water series (N2 and ST regimes); by the time of first-feeding they gained ~45% of dd; and by the fry transition–~65% (**S4 Table**).

Fitting of the experimental data obtained for DV with the Arrhenius model resulted in A = (8.57 ± 4.99) 10^19^ units and Ea = (1.16 ± 0.01) 10^5^ kJ mol^-1^ for D(τ) calculation. In contrast to the dd method, our approach brought D = 1.0 for DV incubated at both ~2.9 and 0.1°C (**S4 Table**). Thus, we may conclude that the proposed approach tends to be less bias-prone than the direct dd method because of accelerated development at low temperatures.

#### Growth rate

Prolonged embryogenesis was found to result in a significant increase of DV embryos hatched in the cold-water series (DV, W and L regimes) as compared to the warm-water ones (N2 and ST regimes), ANOVA for FL: F_5;88_ = 3.6 *P* = 0.050 (**S5 Table** for pairwise comparisons). By the alevin stage, the size gap between cold- and warm-water series retained, F_5;86_ = 10.8 *P* = 0.005. Further, the temperature regimes got aligned across the series due to the summer warming at the spawning grounds. As a result, all DV series continued to grow with a similar rate (F_5;87_ = 11.0 *P* = 0.004), and maintained FL discrepancy at the end of the experiment (F_5;84_ = 12.7 *P* = 0.001). It may be concluded then that under the temperature regimes specific to different LK morphs, DV did not display any serious somatic growth variations (**S9 Fig**). The ‘FL–W’ ratio was the same in DV throughout the series, and the differences in weight between the groups were fully consistent with FL differences.

### LK morphs developmental rate variability

We analyzed the developmental rate of different charrs in terms of D(τ) (**Table 1**). To do so the highest obtained D-value was referred to as a delay in development, while the smallest one–as a relative acceleration of the development. Under natural regimes, W, N1g and N2 morphs demonstrated roughly similar developmental rate close to the DV one; L morph appeared to be dramatically decelerated, while N3 morph displayed an accelerated developmental rate as compared to DV. Under ST regime, most of embryogenesis processes proceeded in warmer conditions rather than under the natural regimes. As a result, somewhat earlier hatching was observed under the natural conditions. From the alevin stage, ST regime became comparatively cooler. Therefore, W and L morphs under ST regime began to accelerate their development compared with the fish reared under the natural regime. Confrontation of the data collected from both experiments revealed that temperature variation had a more pronounced effect on the nosed morphs’ than on W, L morphs and DV. The Spearman ρ for ‘dd(τ)–D(τ)’ ratio (**Table 1**) were: N2 - 0.980; N3 - 0.934; N1g - 0.921; L - 0.846; DV - 0.837; and W - 0.817 (with *P* < 0.001 for all morphs).

**Table 1:** Values of D(τ) and dd (in brackets) for the experimentally reared charrs during 50% transition to the next developmental stage.

Group	Stages						
	eyed egg	free embryo	late embryo	alevin	late alevin	Fry	late fry
Natural temperatures							
DV	0.41 (99)	0.99 (109)	1.09 (121)	1.39 (225)	1.67 (339)	2.08 (506)	3.50 (945)
W	0.39 (137)	0.94 (154)	1.01 (157)	1.29 (194)	1.63 (314)	2.02 (480)	3.44 (1030)
L	0.42 (125)	1.00 (201)	1.09 (219)	1.41 (286)	1.78 (394)	2.33 (621)	3.92 (1055)
N1g	0.41 (147)	0.98 (281)	1.06 (299)	1.39 (386)	1.71 (503)	2.06 (639)	3.49 (1189)
N2	0.41 (166)	0.98 (362)	1.06 (383)	1.39 (489)	1.72 (598)	2.08 (728)	3.51 (1307)
N3	0.39 (129)	0.93 (267)	1.00 (283)	1.27 (346)	1.56 (425)	1.92 (544)	3.28 (1027)
Standard temperature							
DV	0.40 (151)	1.00 (382)	1.08 (411)	1.39 (532)	1.68 (637)	2.10 (799)	3.51 (1305)
W	0.38 (138)	0.93 (352)	0.99 (373)	1.27 (465)	1.52 (560)	1.91 (720)	no data
L	0.40 (152)	1.00 (380)	1.08 (413)	1.38 (525)	1.68 (640)	2.09 (840)	
N1g	0.39 (148)	0.97 (378)	1.04 (405)	1.35 (515)	1.65 (628)	2.06 (780)	
N2	0.40 (149)	0.98 (380)	1.06 (409)	1.36 (517)	1.67 (630)	2.07 (782)	
N3	0.38 (141)	0.94 (353)	1.00 (375)	1.25 (465)	1.56 (579)	1.93 (725)	

### LK morphs growth rate variability

**Fig 3:**
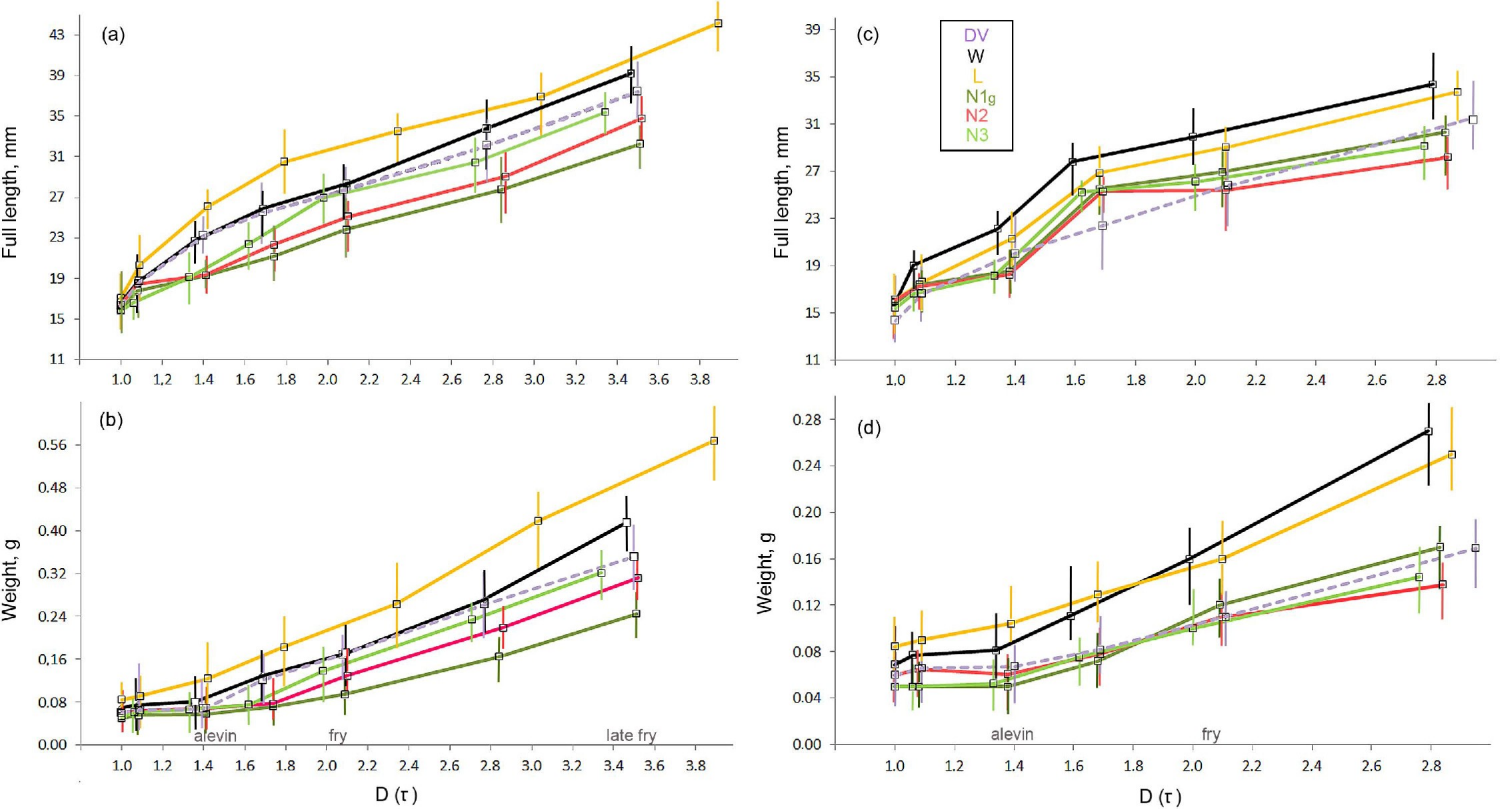
Postnatal somatic growth of the Lake Kronotskoe charr morphs and Dolly Varden reared under the natural (**a**, **b**) and standard (**c**, **d**) temperatures. Mean and min-max values are presented; the morphs are shown in different colors.

We analyzed growth rate in terms of fish length and weight increments measured between the stages singled out according to the parameters of morphology and behavioral traits (**S5 Fig** and **S1 Table**). Under natural regimes, free embryo length (FL) and weight (W) were morph-specific (ANOVA for FL/W: F_5;86_ = 3.7/9.2 *P* = 0.045/0.022; **S6-1 Table** for pairwise comparisons) and depended on the egg size. The newly hatched L and W morphs were significantly larger than the nosed morphs, while DV free embryo exhibited intermediate characteristics. Then throughout the rest of the experiment, W morph demonstrated a steady growth with slight acceleration at the fry stage as compared to DV (**Fig 3A and 3B**; morphs’ sizes at the critical developmental stages are present in **S7 Table**). L morph significantly outstripped other morphs in size up to the alevin stage (F_5;83_ = 30.4/24.4 *P* < 0.001). In contrast, the nosed morphs manifested a markedly slower growth at the embryonic stages. N3 morph accelerated growth by the alevin stage and reached the fry’s size with the size compatible to DV fry size (F_5;83_ = 39.9/57.6 *P* ≥ 0.746). Both remaining nosed morphs, N1g and N2 grew slowly and were smaller than DV even at the late fry stage (F_5;82_ = 43.4/60.8 *P* < 0.001).

Under the specific temperature regimes, the similarity/dissimilarity between DV and the LK morphs growth became especially pronounced (**S10 Fig**). W and N3 morphs’ growth rate coincided with that of DV (pairwise t-test at the final experimental point for FL/W: *P* = 0.952/0.812 and = 0.144/0.218, respectively). L morph grew significantly faster (*P* = 0.022/0.005), whereas N2 morph grew slightly slower (*P* = 0.043/0.050), and N1g one - much slower (*P* = 0.001/<0.001).

A relatively warm ST regime differently affected the growth rate of the LK morphs (**S11 Fig**). The embryos were shorter and possessed a larger yolk as compared to the fish hatched under the natural (cooler) temperatures. L and W morphs’ embryos were of the same size but significantly heavier than the nosed morphs’ embryos (*P* ≤ 0.015). Generally, W and L morphs grew significantly faster than the others, considering their size at the given stage (**S6-2 Table** for pairwise comparisons). Such a variation in size among the morphs was relatively less pronounced than in the experiment with the natural temperatures (**Fig 3C** and **3D**): ANOVA outputs for FL/W at the free embryo stage: F_5;85_ = 3.9/8.4 *P* = 0.050/0.039, at the alevin stage: F_5;85_ = 30.7/31.6 *P* < 0.001, and at the fry stage: F_5;82_ = 12.6/12.1 *P* = 0.003. Different relative positions of W and L morphs on the graphs FL and W increments is explained by a different condition index, which was higher in L morph throughout early ontogeny (**S12 Fig**).

Dissimilarities in the growth rate observed between the fish reared under natural and ST regimes were assessed using the Spearman ρ for ‘dd(τ)–FL(τ)’. It was found that the smallest values indicating a low level of correlation between somatic growth and temperature were obtained for DV (0.778). The highest values evidencing a strong correlation were typical of the nosed morphs (N2 - 0.970, N1g - 0.929 and N3 - 0.908); t intermediate values characterized L (0.840) and W (0.886) morphs (*P* < 0.001 for all morphs). The comparison of the LK charrs reared under natural and ST regimes revealed that somatic growth rate inversely depend on the temperature, i.e. the fish size at the given stage used to increase as the rearing temperature went down (more pronounced in L and W morphs; **S11 Fig**). Meanwhile, the fish reared under diverging regimes did not display any significant FL difference at the end of the experiments (t-test *P* > 0.05).

### Phenology and seasonal developmental shifts

**Fig 4:**
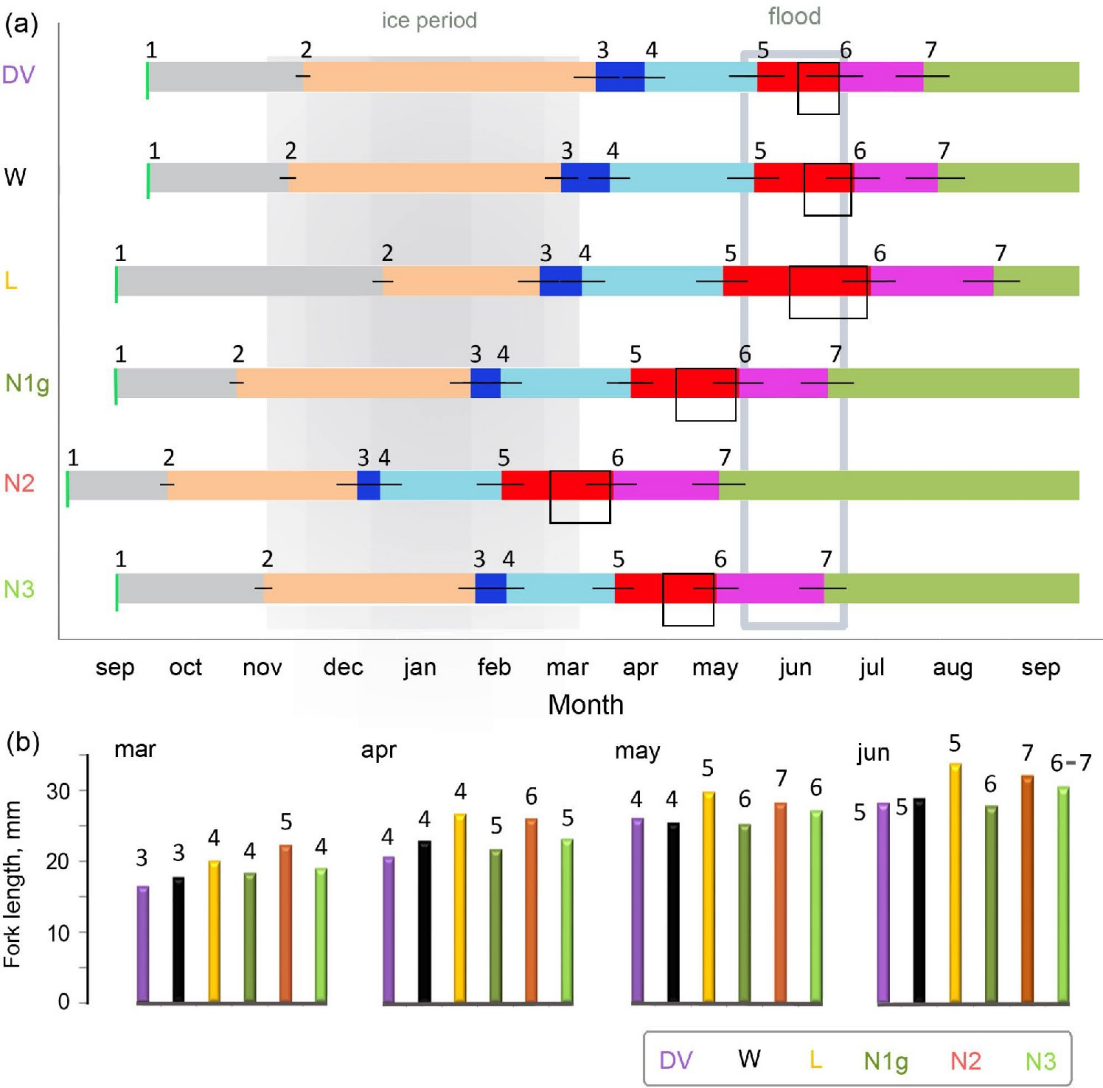
Timing of sequential developmental stage transition in the first year of life (**a**; lines indicate time limits of the developmental stage change, boxes stand for a probable time of leaving the redds = 50% yolk suck desorption at starvation) and early ontogeny linear growth (**b**; the morphs are shown in different colors) of the Lake Kronotskoe charr morphs and Dolly Varden based on the data obtained from the experimental rearing. The numbers mean: 1 –fertilization (peak of spawning), 2 –embryo eye pigmentation, 3 –free embryo (hatching), 4 –late embryo, 5 –alevin (and the start of feeding in the experimental conditions), 6 –late alevin, 7 –fry.

Having superimposed the data on rearing the fish under natural regime on the calendar (**Fig 4A**), we decided to divide the charrs into three distinct groups: those with the delayed development (in terms of real time) development - typical of DV, W and L morphs (≥ 160 days before hatching, ≥ 225 days before external feeding), intermediate development - inherent in N1g and N3 morphs (≤ 135 and 200 days, respectively), and accelerated development - characteristic of N2 morph (≤ 116 and 173 days).

According to our data, DV hatches in March and leaves the redds in the second half of June, which allows them to settle the river habitats in July during the maximum summer water warming. W and L morphs hatch a little earlier, at the end of the ice period, but leave the redds at the same time as DV does, i.e. during the end of the flood (**Fig 4A**). Our experiments clearly demonstrate that in March, the newly hatched DV is significantly smaller than L morph (ANOVA F_5;83_ = 30.4 *P* = 0.001) and does not significantly differ in size from W morph (*P* = 0.072). L morph size exceedance remains till June (F_5;89_ = 42.1 *P* < 0.001; **Fig 4B**).

Phenology of N1g and N3 morphs is practically identical. Both hatch in the depth of winter, leave the redds to the beginning of May before the flood and pass a transition to fry in June at the end of the flood (**Fig 4A**). In all seasons, N1g and N3 morphs do not demonstrate significant differences in size as compared to one another and DV–W morphs (**Fig 4B**), so they are also significantly smaller than L morph (*P* < 0.013).

N2 morph hatches even before the middle of the ice period, leaves the redds in March, and reaches the fry stage before the flood (**Fig 4A**). In March, it is significantly larger (*P* < 0.022) than all the other morphs due to the longer postnatal lifetime. To June, N2 retains the size gap with DV-W-N1g morphs (*P* < 0.009) and is concede only to L morph (*P* = 0.011) (**Fig 4B**).

## Discussion

Our research provided a solid premise to put forward the hypothesis that temperature produces a crucial impact on the LK charr diversification process. Many a study demonstrated the strongest impact of temperature on the developmental and growth rates in sympatric fish [5,33–36,58], contributing to their eco-morphological specialization [59,60]. In salmonids–definitely inclined to the reproduction sites with the preferred (ecologically optimal) temperatures [61–63]–even minor temperature difference occurred at early ontogeny quite suffices to trigger phenotypic divergence [59,64,65]. Under these considerations, it is temperature that seems a vital parameter contributing to the LK charrs diversification.

The LK morphs spawn at remote sites and subsequently develop under specific temperatures, which differ from each other and from the ancestral ones–those specific to DV. The strongest temperature discrepancies between LK morphs’ spawning sites is observed in winter, precisely at the prenatal and early postnatal periods–the most temperature-dependent stages in salmonids [50,66–68]. During this season, the groundwater outlets exhibit a comparatively high water temperature in the tributaries’ middle course [41], the benthivorous N-morphs’ spawning zone. Simultaneously, in the upper courses–the zone of facultative predatory W morph reproduction–the water is strongly affected by atmospheric cooling resulting in an extremely low temperature. The headwaters, preferred by the obligatory piscivorous L morph, remain completely snow-covered, which insulates them from atmospheric cooling and yields a higher average winter temperature as compared to that at W morphs’ reproductive sites in the upper courses [69]. Thus, early stages of the nosed morphs development proceeds under the relatively warm conditions, W morph develops under overcooled conditions, and L morph goes through a prolonged winter period in mountain headwaters. Noteworthily, temperature dynamics at the W morph’s spawning sites shows no drastic difference as compared to the dynamics specific of the ancestral DV.

The results of our experiment on natural temperatures superimposed on the calendar revealed significant differences in the morphs’ hatching and first feeding timing. The predatory W and L morphs replicate the ancestral strategy: they skip the flood period in the spawning ground and implement their primary settlement during intensive water heating in summer. Benthivorous N-morphs hatch a month later from the ancestral and predatory morphs, in the middle of winter; and this developmental gap lasts till the moment of their primary settlement. Though nothing as yet is known on how and when LK charrs occupy the lacustrine niches, we succeeded to collect the overwintered juveniles of predatory morphs in the vicinity of their spawning grounds, whereas late alevins and fry of the benthivorous were caught in the lower course downstream from their spawning sites [45]. This fact allows us to suggest the benthivorous morphs to be capable of running *en masse* downstream during the flood, in the first summer of life. Their small-sized fry seem to settle in shallow lake areas and specialize in benthos, the only available appropriate food on the littoral. Predatory morphs overstay flooding at the stage of a free embryo in the spawning ground. Therefore, their fast-growing fry settle in the upper courses of rivers and winter there. We suggest that such a prolonged tributary habitation causes the fry of predatory morphs to run into the lake at a larger size allowing for feeding on larger prey. This migration opens up an opportunity to switch to the piscivory.

**Fig 5:**
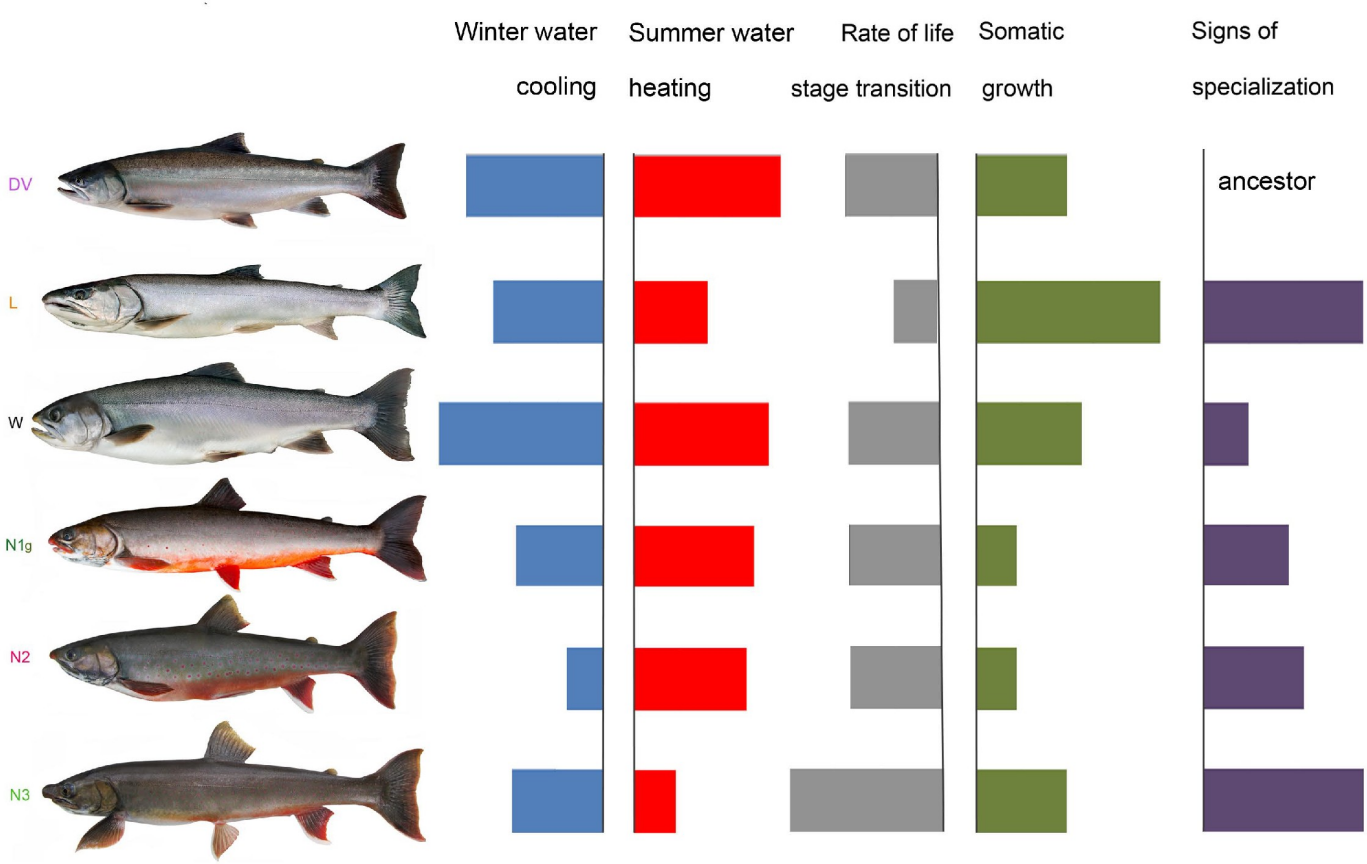
A brief comparison of the developmental temperatures, the rate of life stage transition and the early-life somatic growth among the Lake Kronotskoe charr morphs and the anadromous Dolly Varden. Sector length indicates the trait intensity, as well as overall specialization.

Implementing the D(τ) method allowed us to compare the developmental and growth rates of LK charrs at contrasting temperatures. Under the natural conditions, W morph displays developmental and growth rate mimicking those of the ancestral DV. L morph exhibits an accelerated growth and decelerated development as compared to DV. The development rate of benthivorous N1g and N2 morphs is similar to that of DV, though their growth is slowed, whereas N3 morph shows an accelerated development rate and the growth rate similar to DV (**Fig 5**).

Rearing of DV under different temperature regimes failed to reveal any significant deviations of developmental and growth rates, which indicates a wide temperature tolerance range of the ancestral DV–a feature typical of widely distributed salmonid species [70], provided there are broad landscapes for a disruptive natural selection in contrasting conditions. However, the warmest regimes of rearing (ST and N2) caused a significant reduction of the growth rate at early life stages, though did not strongly diminish the developmental rate. In this regard, the temperature of 2.9⁰C seems to border on the optimal zone for Kamchatkan DV early development.

Both piscivorous morphs reared under ST regime demonstrated a remarkable decrease in the growth rate at the early developmental stages, when ST regime was warmer than the natural temperature. In summer, when ST regime got cooler than the natural temperatures, alevins of piscivorous morphs reared under ST grew relatively faster: the fish size would enlarge at each given stage. Likewise in benthivorous morphs such effects were observed though less pronounced. These findings indicate that the temperature elevation above natural parameters leads to the growth decrease, whereas lowering the temperature stimulates the growth. LK charrs’ efficiency of energy conversion into somatogenesis probably reduces at higher temperatures. The differences in the degree to which LK morphs respond to deviant temperatures suggest their early-life adaptation to narrow temperature ranges.

To some extent, W morph replicates the early developmental dynamics of anadromous DV. However, it seems to be narrowly adapted to low temperatures of embryogenesis and early postnatal growth. L morph demonstrated a slowed-down development, which may be essential for postponing downstream migration. At the same time, L morph exhibited an outstripping growth rate, which should undoubtedly be regarded as an adaptation to an earlier switch to piscivory. Quite evident signs of specialization were also revealed for benthivorous morphs N2 and N3, which are considered to be derivatives from generalistic N1 [71]. The shovelnosed morph (N3) accelerates the development and growth as compared to N1 morph. This tactic compensates for the cold-water developmental conditions and allows for occupying the foraging habitats right on time. To reach the same goal, the second specialized benthivorous morph (N2) shifts spawning to an earlier date in a warm-water stream instead of undertaking developmental acceleration. These two morphs illustrate the well-known scenarios providing for similar adaptive outcomes yet using alternative strategies [72,73].

At later life stages, inverse character of the somatic growth’s dependence on temperature should shift to a direct one. Maturing charrs grow faster at an increasing temperature, as it is the cases with numerous salmonid populations [48,49]. To be noted that the pattern of early ontogeny growth, which the experiment revealed for LK morphs, persists even after maturation (**S13 Fig**). Back-calculation from annual otolith increments demonstrated that piscivorous morphs grow faster than the benthivorous ones throughout their lifespan [74]. Thus, the growth rate should be considered as a highly morph-specific developmental trait.

Let us summarize the key outcomes of this study. Temperature plays a vital role in early ontogeny of sympatric charrs, serving as a master switch that determines the timing of their development and size-weight characteristics. Ecologically different morphs tend to select different spawning sites and exhibit the ability to adapt to the temperature dynamics typical of the selected sites. The differences in temperature dynamics at the reproductive sites underly the developmental and growth rate divergence between the morphs. None of the morphs showed such a broad temperature tolerance as the ancestor, which should be regarded as a consequence of strong disruptive selection that occurred during LK morphs diversification. Therefore, the existence of each morph could be driven by a subtle equilibrium between the specific early developmental program and a timely occupation of the corresponding food niche in the lake under the temperature control.

## Supporting information

S1 Fig Water level dynamics measured at the hydraulic station in the Uzon River mouth (coordinates: 54.728436, 160.009792) during 2013 (black dots) and 2015 (light dots). (DOCX)

S2 Fig The timing of spawning specific to the charr morphs in the Lake Kronotskoe tributaries and to the anadromous Dolly Varden in the Kamchatka River tributaries. The dates of egg collecting are shown as green lines: № 1 –N2, № 2 –N1g, L and N3, № 3 –W and DV. The data is based on annual observations of 2011-2019 on the largest found spawning ground where the eggs were collected for the experiment. The colour intensity denotes the relative density of spawning, i.e. the number of breeding pairs simultaneously accounted at the site. The beginning of spawning was considered as a formation of the first stable spawning pairs holding nests; the height of spawning was the period when dozens of spawners were breeding (no less than 50 pairs per site); the end of spawning was considered as the migration of most spawners downstream. The external appearance and colouration of spawners of each morph is represented below. No ambiguous cases in assigning a fish to one of the morphs were registered at the spawning sites.
(DOCX)

S3 Fig Daily-averaged annual temperature dynamics obtained from the loggers installed into the nests (= redds) of the Lake Kronotskoe charr morphs and anadromous Dolly Varden from the Kamchatka River tributaries. (DOCX)

S4 Fig Normalized distributions of water temperature fluctuations for the most cold- and warm-water morphs during the experimental hatchery in May. Left column - the distribution of the real water temperature around the reference temperature (zero point); right column - the deviations of the measured temperature around the mean line, which reflects the temperature measurement accuracy. The natural temperatures were reproduced in six different water tanks (250 l) using an in-house system. All tanks were placed in the thermostatted room, where the temperature of the air was maintained by Polair SM 232 M cooling system in the range between -3 and +3°C, depending on the season. Each of the water tanks was equipped with the platinum temperature sensor (HoneyWell 700-102BAA-B00) integrated in the bridge measuring scheme. The sensors were placed as close as possible to the eggs or hatched fish. Using the AD7794 (24-bit analog-to-digital converter (Analog Devices) allowed achieving the average noise level of about 0.5 mK. The data were collected by the STM23F103 microcontroller (ST Microsystems) and transmitted to the PC. Besides the temperature measuring unit, each tank was equipped with the water heater, integrated into the water treatment system. The heaters were composed of the standard (133 Ohms per meter) carbon fiber heating wire and the solid-state relay (Crydom D2W) operated by the central STM32 unit. The total power of each heater was 120 W. Each tank slowly (characteristic time of about half-day) cooled due to the interaction with the cold air in the room. The control unit (STM32 + PC) calculated the difference between the target and actual temperature, and the first derivative of the temperature per time. Using these data, the system calculated the amount of heat necessary for temperature maintaining at the given level. The real accuracy of the temperature maintenance was restricted by the periodicity in the air-cooling system (Polair SM 232 M) functioning. The latter had two periods: The short one of about half-hour and the long one of about six hours. The short period (swich-on/switch-off cycles of the air-cooler) caused the temperature fluctuations of about 10 mK, while the defrost procedure performed every six hours caused the overheating of the water by about 30 mK.
(DOCX)

S5 Fig Exterior appearance of the charr developmental (= life) stages under analysis. (DOCX)

S6 Fig Degree-days count of 50% transition to the next developmental stage in the early ontogeny of the Lake Kronotskoe charr morphs and Dolly Varden incubated and reared under imitation of natural temperatures (a) and under the standard temperature conditions (b). 1 –fertilization, 2 –eyed egg, 3 –free embryo (hatching), 4 –late embryo, 5 –alevin (start of feeding in the experimental conditions), 6 –late alevin, 7 –fry, 8 –late fry; lines indicate the time limits of a stage change.
(DOCX)

S7 Fig The channels represented ± Standard Deviation of the daily-averaged annual temperature dynamics in the nests (= redds) of the Lake Kronotskoe charr morphs and the anadromous Dolly Varden. The morphs are shown in different colors. The incut represents the mean values of the Standard Deviation of the daily temperature fluctuations in winter (November 15 to February 15), spring (May 15 to June 14), summer (June 15 to July 31) and autumn (September 15 to October 31) periods specific to each morph and all morphs combined (denotes as ‘mean’). The factorial Multivariate analysis of variance performed in SPSS v.22 (IBM Corp.) determined 3.4 times more powerful effect of the morph identity (F = 1.71, Partial Eta = 0.461) than the logger number (F = 0.32, Partial Eta = 0.135) on the sequential heat accumulation (dd) during spring, summer, autumn and winter at each site. Intercept effect of both factors provides F = 6.30 and *Р* = 0.0067.
(DOCX)

S8 Fig The year dynamics of the first derivative of the water temperature for individual spawning sites of the Lake Kronotskoe charr morphs and the anadromous Dolly Varden. (DOCX)

S9 Fig Postnatal somatic growth of Dolly Varden reared under contrast temperature regimes. Mean and min-max values are presented; the series from different temperatures are shown in different colors.
(DOCX)

S10 Fig Comparison of the linear growth of the Lake Kronotskoe morphs and Dolly Varden (purple colour) in the course of the experiments with the temperature regime typical of the corresponding morphs (and not Dolly Varden). The start point of the curves is normalized to the initial size of the hatched embryo and hatching D = 0. Boxes indicate the sequential stages of development: Free embryo (hatching)–late embryo–alevin (onset of external feeding in the experimental conditions)–fry stage.
(DOCX)

S11 Fig Averaged linear growth of the Lake Kronotskoe charr morphs and Dolly Varden (the morphs are labeled) in the course of the experiments. The comparison of the growth under imitation of natural temperatures (coloured lines) and under the standard temperature (grey lines) is represented. The start point of the curves is normalized to the initial size and D of the hatched embryos. The mean temperatures in two experimental series are shown for the phases of growth separated by vertical lines. Boxes indicate the sequential stages of development: Free embryo (hatching)–late embryo–alevin (onset of external feeding in the experimental conditions)–fry - late fry stage.
(DOCX)

S12 Fig Early ontogeny profiles of Condition index (W ^4^ * FL^-3^) of the Lake Kronotskoe charr morphs and Dolly Varden. The values averaged for the experimental series reared under imitation of natural temperatures and under the standard temperature. The morphs are shown in different colors.
(DOCX)

S13 Fig The annual increase in the fork length of the charrs based on the back-calculation from annual otoliths increments. The data on the Lake Kronotskoe morphs (W, L, N1g, N2, N3) is represented according to Krjevitskaya (2015), the data on the anadromous Dolly Varden growth rate from the Kamchatka River–according to Tiller (2017). Dolly Varden displaying the intermediate growth rate in the experiment is spurting in growth simultaneously with the onset of sea migration. [Krjevitskaya, А.А. 2015. Age and growth of the endemic Dolly Varden morphs, *Salvelinus malma* complex, from Lake Kronotskoe (Eastern Kamchatka). Sochraneniye Bioraznoobraziya Kamchatki i Prilejachich Territoriy. XVI, 279-281 (in Russian); Tiller, I.V. 2017 Biology and population dynamics of the Kamchatkan anadromous Dolly Varden *Salvelinus malma* (Walbaum).KamchatNIRO Publ., Petropavlovsk-Kamchatskiy, RF, 95 pp (in Russian)].
(DOCX)

S1 Table Description of the identified developmental stages. (DOCX)

S2 Table The number of individuals of the Lake Kronotskoe charr morphs and Dolly Varden used for the analysis: Experimental series reared under imitation of natural temperatures/the standard temperature conditions. The developmental points are: 1 –a day after fertilization, 2–50% eyed egg, 3 –free embryo (hatching), 4–50% late embryo, 4a - +0.2 D after 50% late embryo, 5–50% alevin (start of feeding in the experimental conditions), 6–50% late alevin, 7–50% fry, 7a - +0.8 D after 50% fry, 8–50% late fry.
(DOCX)

S3 Table The mean temperatures (°C) of the bottom water level at the spawning grounds of the Lake Kronotskoe charr morphs and the anadromous Dolly Varden. (DOCX)

S4 Table The values of D(τ) and degree-days/days (below the line) at the moments of 50% stages transition for seven experimental series of Dolly Varden reared under contrast temperature regimes. (DOCX)

S5 Table *P*-values of differences from Tukey HSD test complementing ANOVA for the fork length comparison among the Dolly Varden series reared under different temperature regimes: 50% reaching the free embryo (hatching) and alevin (start of feeding in the experimental conditions) stages (above), as well as fry stage (below). Significant differences are marked.
(DOCX)

S6 Table *P*-values of differences from Tukey HSD test complementing ANOVA for the fork length (above)/weight (bellow) comparison among the Lake Kronotskoe charr morphs and the Dolly Varden reared under different temperature regimes, free embryo (hatching)–alevin (start of feeding in the experimental conditions)–fry stage. 1. the natural temperature regimes; 2. the standard temperature regime. Significant differences are marked.
(DOCX)

S7 Table Fork length (mm)/weight (g) of the Lake Kronotskoe charr morphs and Dolly Varden at the moment of 50% transition to the next developmental stage in the experimental series. Mean ± SE and Min–Max values are shown. The live egg diameter is shown instead of FL.
(DOCX)

S1 Data (XLS)
