# Supplementary material for: Ambient temperature as a factor contributing to the developmental divergence in sympatric salmonids
Source: PLoS One. 2021 Oct 15;16(10):e0258536. doi: 10.1371/journal.pone.0258536 (PMC8519426; doi:10.1371/journal.pone.0258536)
Supplement: S1 Fig — (DOCX) [file pone.0258536.s001.docx]

**Supplements**

**Ambient temperature as a factor contributing to the developmental divergence in sympatric salmonids**

Evgeny V. Esin, Grigorii N. Markevich, Nikolai O. Melnik, Dmitriy V. Zlenko & Fedor N. Shkil

**S1** **Fig.** Water level dynamics measured at the hydraulic station in the Uzon River mouth (coordinates: 54.728436, 160.009792) during 2013 (black dots) and 2015 (light dots).
