# Supplementary material for: Ambient temperature as a factor contributing to the developmental divergence in sympatric salmonids
Source: PLoS One. 2021 Oct 15;16(10):e0258536. doi: 10.1371/journal.pone.0258536 (PMC8519426; doi:10.1371/journal.pone.0258536)
Supplement: S2 Fig — The dates of egg collecting are shown as green lines: № 1 –N2, № 2 –N1g, L and N3, № 3 –W and DV. The data is based on annual observations of 2011-2019 on the largest found spawning ground where the eggs were collected for the experiment. The colour intensity denotes the relative density of spawning, i.e. the number of breeding pairs simultaneously accounted at the site. The beginning of spawning was considered as a formation of the first stable spawning pairs holding nests; the height of spawning was the period when dozens of spawners were breeding (no less than 50 pairs per site); the end of spawning was considered as the migration of most spawners downstream. The external appearance and colouration of spawners of each morph is represented below. No ambiguous cases in assigning a fish to one of the morphs were registered at the spawning sites. (DOCX) [file pone.0258536.s002.docx]

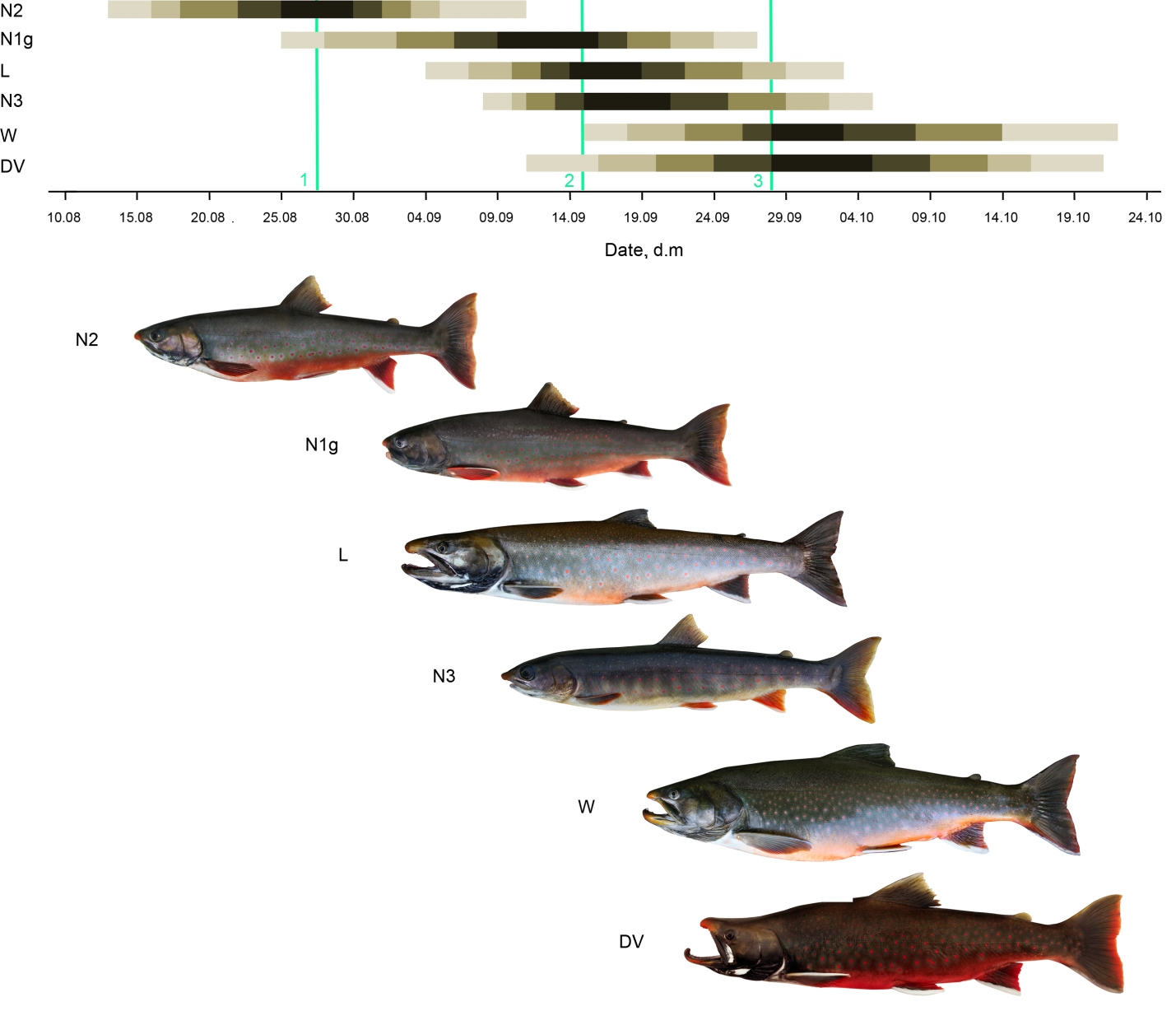


**S2 Fig.** The timing of spawning specific to the charr morphs in the Lake Kronotskoe tributaries and to the anadromous Dolly Varden in the Kamchatka River tributaries. The dates of egg collecting are shown as green lines: № 1 – N2, № 2 – N1g, L and N3, № 3 – W and DV.

The data is based on annual observations of 2011‑2019 on the largest found spawning ground where the eggs were collected for the experiment. The colour intensity denotes the relative density of spawning, i.e. the number of breeding pairs simultaneously accounted at the site. The beginning of spawning was considered as a formation of the first stable spawning pairs holding nests; the height of spawning was the period when dozens of spawners were breeding (no less than 50 pairs per site); the end of spawning was considered as the migration of most spawners downstream.

The external appearance and colouration of spawners of each morph is represented below. No ambiguous cases in assigning a fish to one of the morphs were registered at the spawning sites.
