# Supplementary material for: Ambient temperature as a factor contributing to the developmental divergence in sympatric salmonids
Source: PLoS One. 2021 Oct 15;16(10):e0258536. doi: 10.1371/journal.pone.0258536 (PMC8519426; doi:10.1371/journal.pone.0258536)
Supplement: S3 Fig — (DOCX) [file pone.0258536.s003.docx]

**S3 Fig**. Daily-averaged annual temperature dynamics obtained from the loggers installed into the nests (=redds) of the Lake Kronotskoe charr morphs and anadromous Dolly Varden from the Kamchatka River tributaries.
