# Supplementary material for: Ambient temperature as a factor contributing to the developmental divergence in sympatric salmonids
Source: PLoS One. 2021 Oct 15;16(10):e0258536. doi: 10.1371/journal.pone.0258536 (PMC8519426; doi:10.1371/journal.pone.0258536)
Supplement: S4 Fig — Left column - the distribution of the real water temperature around the reference temperature (zero point); right column - the deviations of the measured temperature around the mean line, which reflects the temperature measurement accuracy. The natural temperatures were reproduced in six different water tanks (250 l) using an in-house system. All tanks were placed in the thermostatted room, where the temperature of the air was maintained by Polair SM 232 M cooling system in the range between -3 and +3°C, depending on the season. Each of the water tanks was equipped with the platinum temperature sensor (HoneyWell 700-102BAA-B00) integrated in the bridge measuring scheme. The sensors were placed as close as possible to the eggs or hatched fish. Using the AD7794 (24-bit analog-to-digital converter (Analog Devices) allowed achieving the average noise level of about 0.5 mK. The data were collected by the STM23F103 microcontroller (ST Microsystems) and transmitted to the PC. Besides the temperature measuring unit, each tank was equipped with the water heater, integrated into the water treatment system. The heaters were composed of the standard (133 Ohms per meter) carbon fiber heating wire and the solid-state relay (Crydom D2W) operated by the central STM32 unit. The total power of each heater was 120 W. Each tank slowly (characteristic time of about half-day) cooled due to the interaction with the cold air in the room. The control unit (STM32 + PC) calculated the difference between the target and actual temperature, and the first derivative of the temperature per time. Using these data, the system calculated the amount of heat necessary for temperature maintaining at the given level. The real accuracy of the temperature maintenance was restricted by the periodicity in the air-cooling system (Polair SM 232 M) functioning. The latter had two periods: The short one of about half-hour and the long one of about six hours. The short period (swich-on/switch-off cycles of the ai [file pone.0258536.s004.docx]

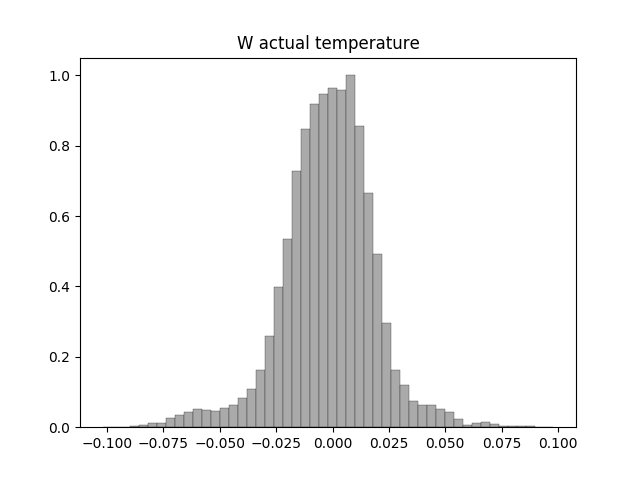

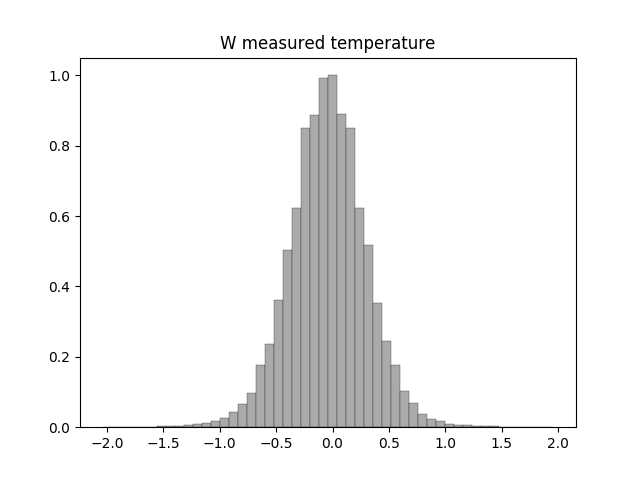


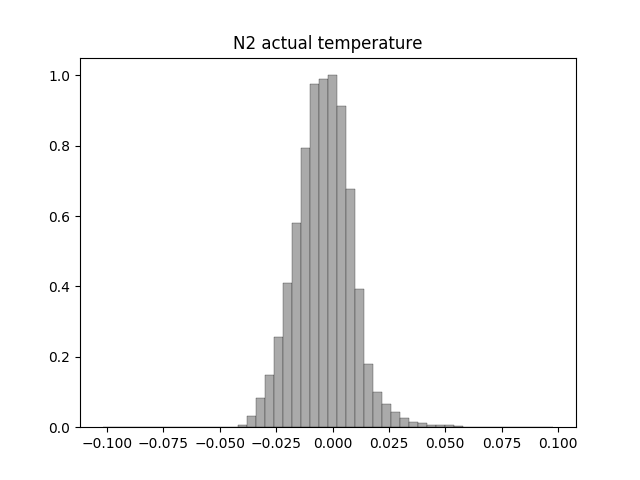

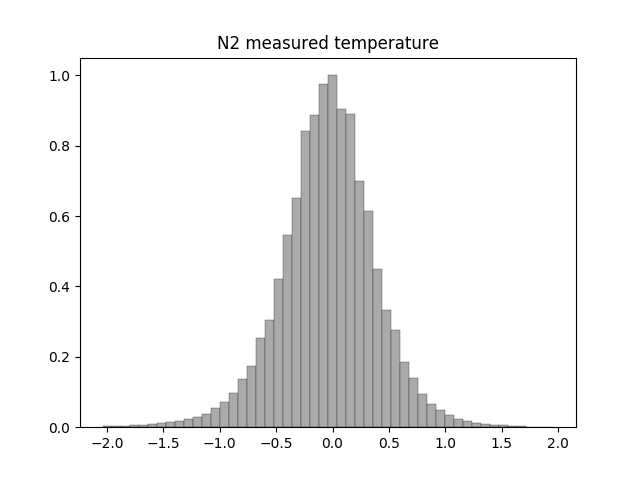


Temperature deviations, ͦC Temperature deviations, x10 ͦC

**S4 Fig.** Normalized distributions of water temperature fluctuations for the most cold- and warm-water morphs during the experimental hatchery in May. Left column ‑ the distribution of the real water temperature around the reference temperature (zero point); right column ‑ the deviations of the measured temperature around the mean line, which reflects the temperature measurement accuracy.

The natural temperatures were reproduced in six different water tanks (250 l) using an in-house system. All tanks were placed in the thermostatted room, where the temperature of the air was maintained by Polair SM 232 M cooling system in the range between -3 and +3^o^C, depending on the season. Each of the water tanks was equipped with the platinum temperature sensor (HoneyWell 700-102BAA-B00) integrated in the bridge measuring scheme. The sensors were placed as close as possible to the eggs or hatched fish. Using the AD7794 (24-bit analog-to-digital converter (Analog Devices) allowed achieving the average noise level of about 0.5 mK. The data were collected by the STM23F103 microcontroller (ST Microsystems) and transmitted to the PC. Besides the temperature measuring unit, each tank was equipped with the water heater, integrated into the water treatment system. The heaters were composed of the standard (133 Ohms per meter) carbon fiber heating wire and the solid-state relay (Crydom D2W) operated by the central STM32 unit. The total power of each heater was 120 W.

Each tank slowly (characteristic time of about half-day) cooled due to the interaction with the cold air in the room. The control unit (STM32 + PC) calculated the difference between the target and actual temperature, and the first derivative of the temperature per time. Using these data, the system calculated the amount of heat necessary for temperature maintaining at the given level.

The real accuracy of the temperature maintenance was restricted by the periodicity in the air-cooling system (Polair SM 232 M) functioning. The latter had two periods: the short one of about half-hour and the long one of about six hours. The short period (swich-on/switch-off cycles of the air-cooler) caused the temperature fluctuations of about 10 mK, while the defrost procedure performed every six hours caused the overheating of the water by about 30 mK.
