# Supplementary material for: Ambient temperature as a factor contributing to the developmental divergence in sympatric salmonids
Source: PLoS One. 2021 Oct 15;16(10):e0258536. doi: 10.1371/journal.pone.0258536 (PMC8519426; doi:10.1371/journal.pone.0258536)
Supplement: S5 Fig — (DOCX) [file pone.0258536.s005.docx]

**S5** **Fig.** Exterior appearance of the charr developmental (= life) stages under analysis.
