# Supplementary material for: Ambient temperature as a factor contributing to the developmental divergence in sympatric salmonids
Source: PLoS One. 2021 Oct 15;16(10):e0258536. doi: 10.1371/journal.pone.0258536 (PMC8519426; doi:10.1371/journal.pone.0258536)
Supplement: S6 Fig — Degree-days count of 50% transition to the next developmental stage in the early ontogeny of the Lake Kronotskoe charr morphs and Dolly Varden incubated and reared under imitation of natural temperatures (a) and under the standard temperature conditions (b). 1 –fertilization, 2 –eyed egg, 3 –free embryo (hatching), 4 –late embryo, 5 –alevin (start of feeding in the experimental conditions), 6 –late alevin, 7 –fry, 8 –late fry; lines indicate the time limits of a stage change. (DOCX) [file pone.0258536.s006.docx]

**S6** **Fig.** Degree-days count of 50% transition to the next developmental stage in the early ontogeny of the Lake Kronotskoe charr morphs and Dolly Varden incubated and reared under imitation of natural temperatures (a) and under the standard temperature conditions (b).

1 – fertilization, 2 – eyed egg, 3 – free embryo (hatching), 4 – late embryo, 5 – alevin (start of feeding in the experimental conditions), 6 – late alevin, 7 – fry, 8 – late fry; lines indicate the time limits of a stage change.
