# Supplementary material for: Ambient temperature as a factor contributing to the developmental divergence in sympatric salmonids
Source: PLoS One. 2021 Oct 15;16(10):e0258536. doi: 10.1371/journal.pone.0258536 (PMC8519426; doi:10.1371/journal.pone.0258536)
Supplement: S7 Fig — The morphs are shown in different colors. The incut represents the mean values of the Standard Deviation of the daily temperature fluctuations in winter (November 15 to February 15), spring (May 15 to June 14), summer (June 15 to July 31) and autumn (September 15 to October 31) periods specific to each morph and all morphs combined (denotes as ‘mean’). The factorial Multivariate analysis of variance performed in SPSS v.22 (IBM Corp.) determined 3.4 times more powerful effect of the morph identity (F = 1.71, Partial Eta = 0.461) than the logger number (F = 0.32, Partial Eta = 0.135) on the sequential heat accumulation (dd) during spring, summer, autumn and winter at each site. Intercept effect of both factors provides F = 6.30 and Р = 0.0067. (DOCX) [file pone.0258536.s007.docx]

**S7 Fig.** The channels represented ± Standard Deviation of the daily-averaged annual temperature dynamics in the nests (= redds) of the Lake Kronotskoe charr morphs and the anadromous Dolly Varden. The morphs are shown in different colors.

The incut represents the mean values of the Standard Deviation of the daily temperature fluctuations in winter (November 15 to February 15), spring (May 15 to June 14), summer (June 15 to July 31) and autumn (September 15 to October 31) periods specific to each morph and all morphs combined (denotes as ‘mean’).

The factorial Multivariate analysis of variance performed in SPSS v.22 (IBM Corp.) determined 3.4 times more powerful effect of the morph identity (F = 1.71, Partial Eta = 0.461) than the logger number (F = 0.32, Partial Eta = 0.135) on the sequential heat accumulation (dd) during spring, summer, autumn and winter at each site. Intercept effect of both factors provides F = 6.30 and *Р* =0.0067.
