# Supplementary material for: Ambient temperature as a factor contributing to the developmental divergence in sympatric salmonids
Source: PLoS One. 2021 Oct 15;16(10):e0258536. doi: 10.1371/journal.pone.0258536 (PMC8519426; doi:10.1371/journal.pone.0258536)
Supplement: S8 Fig — (DOCX) [file pone.0258536.s008.docx]

**S8 Fig.** The year dynamics of the first derivative of the water temperature for individual spawning sites of the Lake Kronotskoe charr morphs and the anadromous Dolly Varden.s
