# Supplementary material for: Ambient temperature as a factor contributing to the developmental divergence in sympatric salmonids
Source: PLoS One. 2021 Oct 15;16(10):e0258536. doi: 10.1371/journal.pone.0258536 (PMC8519426; doi:10.1371/journal.pone.0258536)
Supplement: S9 Fig — Mean and min-max values are presented; the series from different temperatures are shown in different colors. (DOCX) [file pone.0258536.s009.docx]

**S9 Fig.** Postnatal somatic growth of Dolly Varden reared under contrast temperature regimes. Mean and min‑max values are presented; the series from different temperatures are shown in different colors.
