# Supplementary material for: Ambient temperature as a factor contributing to the developmental divergence in sympatric salmonids
Source: PLoS One. 2021 Oct 15;16(10):e0258536. doi: 10.1371/journal.pone.0258536 (PMC8519426; doi:10.1371/journal.pone.0258536)
Supplement: S10 Fig — The start point of the curves is normalized to the initial size of the hatched embryo and hatching D = 0. Boxes indicate the sequential stages of development: Free embryo (hatching)–late embryo–alevin (onset of external feeding in the experimental conditions)–fry stage. (DOCX) [file pone.0258536.s010.docx]

**S10 Fig.** Comparison of the linear growth of the Lake Kronotskoe morphs and Dolly Varden (purple colour) in the course of the experiments with the temperature regime typical of the corresponding morphs (and not Dolly Varden). The start point of the curves is normalized to the initial size of the hatched embryo and hatching D = 0.

Boxes indicate the sequential stages of development: free embryo (hatching) – late embryo – alevin (onset of external feeding in the experimental conditions) – fry stage.
