# Supplementary material for: Ambient temperature as a factor contributing to the developmental divergence in sympatric salmonids
Source: PLoS One. 2021 Oct 15;16(10):e0258536. doi: 10.1371/journal.pone.0258536 (PMC8519426; doi:10.1371/journal.pone.0258536)
Supplement: S11 Fig — The comparison of the growth under imitation of natural temperatures (coloured lines) and under the standard temperature (grey lines) is represented. The start point of the curves is normalized to the initial size and D of the hatched embryos. The mean temperatures in two experimental series are shown for the phases of growth separated by vertical lines. Boxes indicate the sequential stages of development: Free embryo (hatching)–late embryo–alevin (onset of external feeding in the experimental conditions)–fry - late fry stage. (DOCX) [file pone.0258536.s011.docx]

**S11 Fig.** Averaged linear growth of the Lake Kronotskoe charr morphs and Dolly Varden (the morphs are labeled) in the course of the experiments. The comparison of the growth under imitation of natural temperatures (coloured lines) and under the standard temperature (grey lines) is represented. The start point of the curves is normalized to the initial size and D of the hatched embryos. The mean temperatures in two experimental series are shown for the phases of growth separated by vertical lines.

Boxes indicate the sequential stages of development: free embryo (hatching) – late embryo – alevin (onset of external feeding in the experimental conditions) – fry ‑ late fry stage.
