# Supplementary material for: Ambient temperature as a factor contributing to the developmental divergence in sympatric salmonids
Source: PLoS One. 2021 Oct 15;16(10):e0258536. doi: 10.1371/journal.pone.0258536 (PMC8519426; doi:10.1371/journal.pone.0258536)
Supplement: S12 Fig — The values averaged for the experimental series reared under imitation of natural temperatures and under the standard temperature. The morphs are shown in different colors. (DOCX) [file pone.0258536.s012.docx]

**S12** **Fig.** Early ontogeny profiles of Condition index (W ^4^ _*_ FL^‑3^) of the Lake Kronotskoe charr morphs and Dolly Varden. The values averaged for the experimental series reared under imitation of natural temperatures and under the standard temperature. The morphs are shown in different colors.
