# Supplementary material for: Ambient temperature as a factor contributing to the developmental divergence in sympatric salmonids
Source: PLoS One. 2021 Oct 15;16(10):e0258536. doi: 10.1371/journal.pone.0258536 (PMC8519426; doi:10.1371/journal.pone.0258536)
Supplement: S13 Fig — The data on the Lake Kronotskoe morphs (W, L, N1g, N2, N3) is represented according to Krjevitskaya (2015), the data on the anadromous Dolly Varden growth rate from the Kamchatka River–according to Tiller (2017). Dolly Varden displaying the intermediate growth rate in the experiment is spurting in growth simultaneously with the onset of sea migration. [Krjevitskaya, А.А. 2015. Age and growth of the endemic Dolly Varden morphs, Salvelinus malma complex, from Lake Kronotskoe (Eastern Kamchatka). Sochraneniye Bioraznoobraziya Kamchatki i Prilejachich Territoriy. XVI, 279-281 (in Russian); Tiller, I.V. 2017 Biology and population dynamics of the Kamchatkan anadromous Dolly Varden Salvelinus malma (Walbaum).KamchatNIRO Publ., Petropavlovsk-Kamchatskiy, RF, 95 pp (in Russian)]. (DOCX) [file pone.0258536.s013.docx]

**S13** **Fig.** The annual increase in the fork length of the charrs based on the back-calculation from annual otoliths increments. The data on the Lake Kronotskoe morphs (W, L, N1g, N2, N3) is represented according to Krjevitskaya (2015), the data on the anadromous Dolly Varden growth rate from the Kamchatka River –according to Tiller (2017). Dolly Varden displaying the intermediate growth rate in the experiment is spurting in growth simultaneously with the onset of sea migration.

Krjevitskaya, А.А. 2015. Age and growth of the endemic Dolly Varden morphs, *Salvelinus malma* complex, from Lake Kronotskoe (Eastern Kamchatka). Sochraneniye Bioraznoobraziya Kamchatki i Prilejachich Territoriy. XVI, 279‑281 (in Russian).

Tiller, I.V. 2017 Biology and population dynamics of the Kamchatkan anadromous Dolly Varden *Salvelinus malma* (Walbaum).KamchatNIRO Publ., Petropavlovsk-Kamchatskiy, RF, 95 pp (in Russian).
