# Supplementary material for: Ambient temperature as a factor contributing to the developmental divergence in sympatric salmonids
Source: PLoS One. 2021 Oct 15;16(10):e0258536. doi: 10.1371/journal.pone.0258536 (PMC8519426; doi:10.1371/journal.pone.0258536)
Supplement: S1 Table — (DOCX) [file pone.0258536.s014.docx]

**S1** **Table.** Description of the identified developmental stages.

| Developmental interval | Behavior | Dynamics of anatomical and morphological states | | | |
| --- | --- | --- | --- | --- | --- |
|  |  | fins | craniofacial | yolk sack | colouration |
| Eyed egg | motility develops | fin fold forming | gill arches are formed; notochord begins to vacuolise | vitelline plexus develops | pigment in the optic cups, the blood turns pink |
| Free embryo | passive, lying on the flank | continuous fin fold, ossified rays in the caudal blade | germs of maxilla and dentary (ossified or not) | plumpish shape, the weight is > 50% of that of the organism | melanophore strands on the flanks, ‘pigment cap’ |
| Late free embryo  (late embryo) | active flocking, negative phototaxis | formed posterior edge of the dorsal fin, ossified rays in the dorsal fin | scutes of maxilla and dentary with the first teeth primordia, ossified parasphenoid germ | explanate shape, the weight is ≈ 50% of that of the organism | melanophores aggregate in spots, their density increases |
| Alevin | positive phototaxis, vertical takeoffs and swallowing external food | formed posterior edge of the anal fin, ossification in all fins | ossified premaxilla and lingua | retracted sack, the weight is ≈ 20‑40% of that of the organism | 3‑5 expressed spots |
| Late alevin | swim freely | totally separated dorsal and anal fins, segmentation of the rays in the dorsal and anal fins | ossified basihyal element and hypobranchial elements of the anterior arches, formed walls of the seismosensory channel in the dentary and frontal | the remnants of the sack are covered with body walls, the weight is < 20% of that of organism | 6‑9 expressed spots |
| Fry  (=juvenile) | swim freely | separated adipose fin, total resorption of the fin fold | ossified supraethmoid, orbital series and gill arches | the yolk sack absorption is completed | guanine on operculum, the row of parr marks |
| Late fry  (= ‘parr’) | swim freely | ray branching in all fins | partially accreted teeth on all armored bones instead of the teeth primordia | no yolk sack | silvery flanks, portioned parr marks and squamation of the lateral line and adjacent parts of the body |
