# Supplementary material for: Ambient temperature as a factor contributing to the developmental divergence in sympatric salmonids
Source: PLoS One. 2021 Oct 15;16(10):e0258536. doi: 10.1371/journal.pone.0258536 (PMC8519426; doi:10.1371/journal.pone.0258536)
Supplement: S2 Table — The developmental points are: 1 –a day after fertilization, 2–50% eyed egg, 3 –free embryo (hatching), 4–50% late embryo, 4a - +0.2 D after 50% late embryo, 5–50% alevin (start of feeding in the experimental conditions), 6–50% late alevin, 7–50% fry, 7a - +0.8 D after 50% fry, 8–50% late fry. (DOCX) [file pone.0258536.s015.docx]

**S2 Table.** The number of individuals of the Lake Kronotskoe charr morphs and Dolly Varden used for the analysis: experimental series reared under imitation of natural temperatures / the standard temperature conditions.

| Analysis | Morph | Developmental point | | | | | | | | | |
| --- | --- | --- | --- | --- | --- | --- | --- | --- | --- | --- | --- |
|  |  | 1 | 2 | 3 | 4 | 4a | 5 | 6 | 7 | 7a | 8 |
| Developmental stage account | DV* | >450 | ~ 280/160 | ~ 210/140 | ~ 190/120 | - | ~ 175/90 | ~ 135/70 | ~ 85/30 | - | 35/- |
|  | W | >400 | ~ 240/140 | ~ 180/120 | ~ 160/100 | - | ~ 120/80 | ~ 90/60 | ~ 70/30 | - | 30/- |
|  | L | >400 | ~ 240/140 | ~ 180/120 | ~ 160/100 | - | ~ 120/80 | ~ 90/60 | ~ 70/30 | - | 30/- |
|  | N1g | >400 | ~ 225/140 | ~ 165/120 | ~ 145/100 | - | ~ 115/80 | ~ 85/60 | ~ 65/30 | - | 30/- |
|  | N2 | >400 | ~ 240/140 | ~ 180/120 | ~ 160/100 | - | ~ 120/80 | ~ 90/60 | ~ 70/30 | - | 30/- |
|  | N3 | >400 | ~ 220/135 | ~ 160/115 | ~ 140/100 | - | ~ 110/70 | ~ 80/50 | ~ 60/20 | - | 25/- |
| Fork length (mm) / weight (g) assessment | DV* | - | 20/- | 16/15 | 15/15 | 15/- | 15/15 | 15/15 | 15/15 | 15/15 | 15/- |
|  | W | - | 20/- | 15/15 | 15/15 | 15/- | 15/15 | 15/15 | 15/15 | 15/15 | 15/- |
|  | L | - | 15/- | 15/15 | 15/15 | 15/- | 15/15 | 15/15 | 15/15 | 15/15 | 15/- |
|  | N1g | - | 15/- | 15/15 | 15/15 | 10/- | 13/15 | 13/15 | 13/15 | 10/15 | 12/- |
|  | N2 | - | 15/- | 15/15 | 15/15 | 15/- | 15/15 | 15/15 | 15/15 | 15/15 | 15/- |
|  | N3 | - | 15/- | 15/15 | 15/15 | 10/- | 15/15 | 15/15 | 15/12 | 10/15 | 15/- |

Note. The developmental points are: 1 – a day after fertilization, 2 – 50% eyed egg, 3 – free embryo (hatching), 4 – 50% late embryo, 4a ‑ +0.2 D after 50% late embryo, 5 – 50% alevin (start of feeding in the experimental conditions), 6 – 50% late alevin, 7 – 50% fry, 7a ‑ +0.8 D after 50% fry, 8 – 50% late fry.

* ‑ the same number of individuals as in the experiment with the standard temperature was used for the analysis of DV in the series reared under W, L, N1g and N2 morphs’ temperature regimes.
