# Supplementary material for: Ambient temperature as a factor contributing to the developmental divergence in sympatric salmonids
Source: PLoS One. 2021 Oct 15;16(10):e0258536. doi: 10.1371/journal.pone.0258536 (PMC8519426; doi:10.1371/journal.pone.0258536)
Supplement: S3 Table — (DOCX) [file pone.0258536.s016.docx]

**S3 Table.** The mean temperatures (^o^C) of the bottom water level at the spawning grounds of the Lake Kronotskoe charr morphs and the anadromous Dolly Varden.

| Morph | Incubation period  (period in the redds) | Period from alevin to fry stage | Winter  period,  November 01 - March 31 | Flood period,  May 15 – July 05 | Summer  period  July 05 - September 15 | Mean annual |
| --- | --- | --- | --- | --- | --- | --- |
| DV | 0.71 (1.21) | 3.79 | 0.38 | 3.81 | 5.96 | 2.39 |
| W | 0.66 (1.05) | 3.72 | 0.14 | 3.87 | 5.24 | 2.32 |
| L | 1.19 (1.37) | 3.25 | 0.81 | 2.06 | 3.98 | 2.10 |
| N1g | 2.20 (1.99) | 2.24 | 1.52 | 3.61 | 4.82 | 2.87 |
| N2 | 3.23 (2.81) | 2.82 | 2.32 | 3.56 | 4.17 | 3.22 |
| N3 | 1.83 (1.75) | 2.13 | 1.61 | 3.08 | 3.28 | 2.30 |
