# Supplementary material for: Ambient temperature as a factor contributing to the developmental divergence in sympatric salmonids
Source: PLoS One. 2021 Oct 15;16(10):e0258536. doi: 10.1371/journal.pone.0258536 (PMC8519426; doi:10.1371/journal.pone.0258536)
Supplement: S4 Table — (DOCX) [file pone.0258536.s017.docx]

**S4** **Table.** The values of D(τ) and degree-days / days (below the line) at the moments of 50% stages transition for seven experimental series of Dolly Varden reared under contrast temperature regimes.

| Temperature regime | Stage | | | | | |
| --- | --- | --- | --- | --- | --- | --- |
|  | eyed egg | free embryo | late embryo | alevin | late alevin | fry |
| DV | 0.41  99 / 63 | 0.99  109 / 183 | 1.09  121 / 201 | 1.39  225 / 239 | 1.67  339 / 269 | 2.08  506 / 313 |
| W | 0.41  140 / 62 | 0.99  154 / 189 | 1.09  157 / 202 | 1.39  230 / 241 | 1.68  346 / 273 | 2.09  513 / 303 |
| L | 0.43  127 / 60 | 1.01  207 / 167 | 1.10  223 / 182 | 1.40  286 / 232 | 1.70  370 / 276 | 2.12  536 / 314 |
| N1g | 0.41  148 / 50 | 1.01  287 / 145 | 1.09  310 / 158 | 1.40  392 / 206 | 1.69  492 / 243 | 2.10  662 / 284 |
| N2 | 0.42  173 / 49 | 1.01  373 / 134 | 1.10  398 / 146 | 1.41  496 / 187 | 1.70  595 / 222 | 2.12  750 / 266 |
| N3 | 0.42  136 / 54 | 1.01  286 / 149 | 1.10  306 / 162 | 1.41  384 / 211 | 1.70  469 / 254 | 2.11  616 / 304 |
| Standard | 0.40  151 / 45 | 1.00  382 / 113 | 1.08  411 / 122 | 1.39  532 / 159 | 1.68  637 / 193 | 2.10  799 / 240 |
