# Supplementary material for: Ambient temperature as a factor contributing to the developmental divergence in sympatric salmonids
Source: PLoS One. 2021 Oct 15;16(10):e0258536. doi: 10.1371/journal.pone.0258536 (PMC8519426; doi:10.1371/journal.pone.0258536)
Supplement: S5 Table — Significant differences are marked. (DOCX) [file pone.0258536.s018.docx]

**S5** **Table.** *P*-values of differences from Tukey HSD test complementing ANOVA for the fork length comparison among the Dolly Varden series reared under different temperature regimes: 50% reaching the free embryo (hatching) and alevin (start of feeding in the experimental conditions) stages (above), as well as fry stage (below).

| Temperature regime | DV | W | L | N1g | N2 | N3 | Standard |
| --- | --- | --- | --- | --- | --- | --- | --- |
| DV |  | 0.9004 0.1612 | 0.9997 0.0629 | 0.6707 0.1612 | **0.0474 0.0003** | 0.8321 0.1818 | **0.0013 0.0001** |
| W | 1.000 |  | 0.7460 0.9997 | 0.3097 0.7460 | **0.0413 0.0121** | 0.9004 0.7460 | **0.0011 0.0003** |
| L | 0.2328 | 0.2328 |  | 0.6707 1.000 | **0.0474 0.0084** | 0.6481 0.1531 | **0.0013 0.0003** |
| N1g | 0.1154 | 0.1612 | 0.9997 |  | 0.0874 **0.0084** | 0.3097 0.0851 | **0.0039 0.0003** |
| N2 | 0.2328 | 0.2328 | **0.0013** | **0.0003** |  | **0.0474 0.0127** | **0.0157 0.0127** |
| N3 | 0.2328 | 0.6701 | 0.7451 | 0.0955 | 0.0699 |  | **0.0022 0.0005** |
| Standard | **0.0127** | **0.0003** | **0.0003** | **0.0001** | 0.0629 | **0.0013** |  |

Note. Significant differences are marked.
