# Supplementary material for: Ambient temperature as a factor contributing to the developmental divergence in sympatric salmonids
Source: PLoS One. 2021 Oct 15;16(10):e0258536. doi: 10.1371/journal.pone.0258536 (PMC8519426; doi:10.1371/journal.pone.0258536)
Supplement: S6 Table — 1. the natural temperature regimes; 2. the standard temperature regime. Significant differences are marked. (DOCX) [file pone.0258536.s019.docx]

**S6** **Table.** *P*-values of differences from Tukey HSD test complementing ANOVA for the fork length (above) / weight (bellow) comparison among the Lake Kronotskoe charr morphs and the Dolly Varden reared under different temperature regimes, free embryo (hatching) – alevin (start of feeding in the experimental conditions)– fry stage.

**1.** the natural temperature regimes

| Morph | DV |  |  | W |  |  | L |  |  | N1 |  |  | N2 |  |  | N3 |  |  |
| --- | --- | --- | --- | --- | --- | --- | --- | --- | --- | --- | --- | --- | --- | --- | --- | --- | --- | --- |
| DV |  |  |  | 0.9997 | 0.7108 | 0.2984 | 0.6563 | **0.0013** | **0.0038** | 0.3408 | **0.0013** | **0.0012** | 0.9974 | **0.0013** | **0.0039** | 0.4296 | **0.0013** | 0.1061 |
| W | 0.2287 | 0.8780 | 0.7460 |  |  |  | 0.8523 | **0.0013** | **0.0122** | **0.0480** | **0.0013** | **0.0010** | 0.7723 | **0.0011** | **0.0084** | **0.0387** | **0.0013** | 0.0943 |
| L | **0.0413** | **0.0013** | **0.0001** | 0.1894 | **0.0013** | **0.0013** |  |  |  | **0.0127** | **0.0013** | **0.0012** | **0.0474** | **0.0013** | **0.0014** | **0.0112** | **0.0013** | **0.0012** |
| N1g | 0.3401 | **0.0413** | **0.0001** | **0.0013** | **0.0413** | **0.0012** | **0.0013** | **0.0013** | **0.0012** |  |  |  | 0.5073 | 1.0000 | **0.0412** | 0.9993 | 1.0000 | **0.0025** |
| N2 | 1.0000 | **0.0413** | 0.0629 | **0.0431** | **0.0413** | 0.0621 | **0.0013** | **0.0013** | **0.0012** | 0.2328 | 0.1612 | **0.0250** |  |  |  | 0.6273 | 1.0000 | **0.0138** |
| N3 | 0.1154 | **0.0413** | 0.7149 | **0.0127** | **0.0413** | 0.7460 | **0.0013** | **0.0013** | **0.0012** | 1.0000 | 0.6707 | **0.0048** | 0.3097 | 0.5060 | 0.0532 |  |  |  |

**2.** the standard temperature regime

| Morph | DV |  |  | W |  |  | L |  |  | N1 |  |  | N2 |  |  | N3 |  |  |
| --- | --- | --- | --- | --- | --- | --- | --- | --- | --- | --- | --- | --- | --- | --- | --- | --- | --- | --- |
| DV |  |  |  | **0.0013** | **0.0013** | **0.0042** | **0.0013** | **0.0501** | **0.0126** | **0.0013** | 0.1395 | 0.0515 | **0.0013** | 0.0682 | 0.9704 | 0.0710 | 0.5779 | 0.1825 |
| W | 0.1255 | **0.0013** | **0.0259** |  |  |  | 0.0713 | 0.0579 | 0.0642 | 0.2494 | **0.0013** | 0.0573 | 0.7862 | **0.0013** | **0.0062** | 0.0820 | **0.0010** | **0.0017** |
| L | **0.0013** | **0.0013** | **0.0101** | 0.0734 | 0.0636 | 0.1179 |  |  |  | 0.9995 | **0.0013** | 0.2274 | 0.8125 | **0.0013** | **0.0299** | 0.5341 | **0.0013** | **0.0014** |
| N1g | 0.0992 | 0.4269 | 0.2868 | **0.0040** | **0.0013** | **0.0381** | **0.0013** | **0.0013** | 0.9785 |  |  |  | 0.6192 | 0.3457 | 0.0016 | 0.7075 | 0.5972 | 0.0013 |
| N2 | 0.0598 | 0.3647 | 0.8257 | **0.0151** | **0.0013** | **0.0284** | **0.0015** | **0.0013** | **0.0051** | 0.5922 | 1.0000 | 0.0126 |  |  |  | 0.0782 | 0.3682 | 0.8318 |
| N3 | 0.1388 | 0.4834 | 0.0780 | **0.0009** | **0.0006** | **0.0010** | **0.0013** | **0.0013** | **0.0012** | 1.0000 | 0.0087 | **0.0013** | 0.6624 | 0.0668 | 0.1321 |  |  |  |

Note. Significant differences are marked.
