# Supplementary material for: Ambient temperature as a factor contributing to the developmental divergence in sympatric salmonids
Source: PLoS One. 2021 Oct 15;16(10):e0258536. doi: 10.1371/journal.pone.0258536 (PMC8519426; doi:10.1371/journal.pone.0258536)
Supplement: S7 Table — Mean ± SE and Min–Max values are shown. The live egg diameter is shown instead of FL. (DOCX) [file pone.0258536.s020.docx]

**S7 Table.** Fork length (mm) / weight (g) of the Lake Kronotskoe charr morphs and Dolly Varden at the moment of 50% transition to the next developmental stage in the experimental series. Mean ± SE and Min–Max values are shown.

| Morph | Stage | | | | | | | | | | | | | | | | | | | | | | | | | | |  |
| --- | --- | --- | --- | --- | --- | --- | --- | --- | --- | --- | --- | --- | --- | --- | --- | --- | --- | --- | --- | --- | --- | --- | --- | --- | --- | --- | --- | --- |
|  | eyed egg* | | | | free embryo (hatching) | | | | | | | late embryo | | | | | | | +0.2 D after 50% late embryo | | | | | | |  | |  |
| Imitation of natural temperatures | | | | | | | | | | | | | | | | | | | | | | | | | | | |  |
| DV | 5.1 ± 0.25 | 3.9 | - | 6.5 | | 16.3 | ± | 0.34 | 13.7 | ‑ | 18.1 | | 18.7 | ± | 0.29 | 14.0 | - | 19.9 | | 22.9 | ± | 0.28 | 19.2 | ‑ | 24.3 | | → | |
|  | 0.06 ± 0.005 | 0.04 | - | 0.08 | | 0.06 | ± | 0.004 | 0.04 | ‑ | 0.08 | | 0.07 | ± | 0.003 | 0.05 | - | 0.15 | | 0.07 | ± | 0.002 | 0.06 | ‑ | 0.09 | |  | |
| W | 5.7 ± 0.18 | 5.1 | - | 6.6 | | 16.7 | ± | 0.37 | 14.0 | ‑ | 18.4 | | 18.6 | ± | 0.35 | 14.4 | - | 20.4 | | 22.3 | ± | 0.25 | 20.1 | ‑ | 23.9 | | → | |
|  | 0.06 ± 0.005 | 0.05 | - | 0.08 | | 0.07 | ± | 0.002 | 0.05 | ‑ | 0.08 | | 0.08 | ± | 0.005 | 0.05 | - | 0.13 | | 0.08 | ± | 0.001 | 0.07 | ‑ | 0.11 | |  | |
| L | 6.4 ± 0.15 | 6.0 | - | 6.9 | | 17.1 | ± | 0.15 | 16.2 | ‑ | 18.3 | | 20.3 | ± | 0.28 | 16.2 | - | 23.1 | | 25.8 | ± | 0.26 | 24.2 | ‑ | 27.1 | | → | |
|  | 0.07 ± 0.005 | 0.06 | - | 0.10 | | 0.08 | ± | 0.002 | 0.07 | ‑ | 0.10 | | 0.09 | ± | 0.05 | 0.07 | - | 0.13 | | 0.10 | ± | 0.003 | 0.09 | ‑ | 0.13 | |  | |
| N1g | 4.7 ± 0.25 | 3.9 | - | 5.5 | | 15.9 | ± | 0.44 | 14.0 | ‑ | 17.1 | | 17.6 | ± | 0.38 | 14.3 | - | 19.3 | | 18.8 | ± | 0.42 | 17.9 | ‑ | 19.9 | | → | |
|  | 0.05 ± 0.003 | 0.05 | - | 0.07 | | 0.05 | ± | 0.002 | 0.05 | ‑ | 0.07 | | 0.06 | ± | 0.003 | 0.05 | - | 0.09 | | 0.06 | ± | 0.002 | 0.04 | ‑ | 0.07 | |  | |
| N2 | 5.0 ± 0.25 | 4.3 | - | 5.5 | | 16.4 | ± | 0.10 | 14.9 | ‑ | 17.4 | | 18.5 | ± | 0.26 | 15.0 | - | 19.2 | | 19.0 | ± | 0.30 | 17.0 | ‑ | 21.3 | | → | |
|  | 0.06 ± 0.005 | 0.05 | - | 0.07 | | 0.06 | ± | 0.003 | 0.05 | ‑ | 0.07 | | 0.06 | ± | 0.002 | 0.05 | - | 0.09 | | 0.06 | ± | 0.003 | 0.04 | ‑ | 0.07 | |  | |
| N3 | 4.6 ± 0.25 | 4.1 | - | 5.2 | | 15.8 | ± | 0.26 | 14.8 | ‑ | 18.1 | | 16.6 | ± | 0.25 | 15.0 | - | 18.9 | | 18.7 | ± | 0.25 | 17.0 | ‑ | 19.8 | | → | |
|  | 0.05 ± 0.003 | 0.05 | - | 0.07 | | 0.05 | ± | 0.001 | 0.05 | ‑ | 0.07 | | 0.06 | ± | 0.002 | 0.05 | - | 0.08 | | 0.06 | ± | 0.001 | 0.04 | ‑ | 0.07 | |  | |
| Standard temperature conditions | | | | | | | | | | | | | | | | | | | | | | | | | | | |  |
| DV |  |  |  |  | | 14.3 | ± | 0.14 | 13.3 | ‑ | 15.4 | | 16.6 | ± | 0.29 | 13.9 | ‑ | 18.8 | | 19.1 | ± | 0.33 | 17.9 | ‑ | 22.1 | | → | |
|  |  |  |  |  | | 0.05 | ± | 0.001 | 0.04 | ‑ | 0.07 | | 0.06 | ± | 0.002 | 0.04 | ‑ | 0.07 | | 0.06 | ± | 0.003 | 0.04 | ‑ | 0.07 | |  | |
| W |  |  |  |  | | 15.9 | ± | 0.29 | 12.1 | ‑ | 17.0 | | 19.0 | ± | 0.26 | 16.9 | ‑ | 20.3 | | 21.6 | ± | 0.33 | 20.2 | ‑ | 23.5 | | → | |
|  |  |  |  |  | | 0.07 | ± | 0.005 | 0.04 | ‑ | 0.10 | | 0.08 | ± | 0.003 | 0.04 | ‑ | 0.09 | | 0.08 | ± | 0.005 | 0.07 | ‑ | 0.09 | |  | |
| L |  |  |  |  | | 16.2 | ± | 0.31 | 12.3 | ‑ | 17.1 | | 17.6 | ± | 0.28 | 14.9 | ‑ | 20.2 | | 20.9 | ± | 0.30 | 20.1 | ‑ | 22.3 | | → | |
|  |  |  |  |  | | 0.09 | ± | 0.002 | 0.07 | ‑ | 0.10 | | 0.09 | ± | 0.003 | 0.07 | ‑ | 0.11 | | 0.10 | ± | 0.002 | 0.08 | ‑ | 0.11 | |  | |
| N1g |  |  |  |  | | 15.8 | ± | 0.20 | 14.3 | ‑ | 17.3 | | 17.4 | ± | 0.18 | 15.2 | ‑ | 18.1 | | 18.3 | ± | 0.22 | 17.9 | ‑ | 19.2 | | → | |
|  |  |  |  |  | | 0.05 | ± | 0.002 | 0.05 | ‑ | 0.07 | | 0.05 | ± | 0.001 | 0.05 | ‑ | 0.07 | | 0.05 | ± | 0.002 | 0.04 | ‑ | 0.05 | |  | |
| N2 |  |  |  |  | | 16.1 | ± | 0.18 | 15.5 | ‑ | 17.1 | | 17.2 | ± | 0.47 | 15.7 | ‑ | 18.0 | | 17.8 | ± | 0.26 | 16.8 | ‑ | 19.4 | | → | |
|  |  |  |  |  | | 0.05 | ± | 0.002 | 0.05 | ‑ | 0.07 | | 0.07 | ± | 0.002 | 0.05 | ‑ | 0.07 | | 0.05 | ± | 0.001 | 0.05 | ‑ | 0.05 | |  | |
| N3 |  |  |  |  | | 15.4 | ± | 0.17 | 14.1 | ‑ | 16.5 | | 16.6 | ± | 0.25 | 15.0 | ‑ | 18.0 | | 18.8 | ± | 0.21 | 16.9 | ‑ | 19.4 | | → | |
|  |  |  |  |  | | 0.05 | ± | 0.001 | 0.05 | ‑ | 0.06 | | 0.06 | ± | 0.002 | 0.04 | ‑ | 0.06 | | 0.05 | ± | 0.001 | 0.05 | ‑ | 0.06 | |  | |

**S7 Table** (continuation)

| Morph | | Stage | | | | | | | | | | | | | | | | | | | | | | | | |
| --- | --- | --- | --- | --- | --- | --- | --- | --- | --- | --- | --- | --- | --- | --- | --- | --- | --- | --- | --- | --- | --- | --- | --- | --- | --- | --- |
|  |  |  | alevin (feeding in the experiment) | | | | | | late alevin (primary settlement) | | | | | | fry (resettlement) | | | | | | late fry | | | | | |
|  | Imitation of natural temperatures | | | | | | | | | | | | | | | | | | | | | | | | | |
| DV | | → | 23.2 | ± | 0.30 | 19.8 | ‑ | 25.0 | 25.5 | ± | 0.33 | 20.8 | - | 28.8 | 28.6 | ± | 0.40 | 23.8 | ‑ | 30.8 | 37.4 | ± | 0.33 | 34.7 | ‑ | 42.2 |
|  | |  | 0.07 | ± | 0.002 | 0.06 | ‑ | 0.09 | 0.12 | ± | 0.004 | 0.07 | - | 0.17 | 0.17 | ± | 0.007 | 0.07 | ‑ | 0.21 | 0.35 | ± | 0.107 | 0.30 | ‑ | 0.41 |
| W | | → | 22.7 | ± | 0.21 | 20.6 | ‑ | 23.8 | 25.9 | ± | 0.26 | 23.1 | - | 27.4 | 28.4 | ± | 0.31 | 25.0 | ‑ | 31.1 | 39.5 | ± | 0.25 | 36.9 | ‑ | 44.0 |
|  | |  | 0.08 | ± | 0.001 | 0.07 | ‑ | 0.09 | 0.13 | ± | 0.006 | 0.08 | - | 0.17 | 0.15 | ± | 0.007 | 0.09 | ‑ | 0.20 | 0.37 | ± | 0.009 | 0.31 | ‑ | 0.42 |
| L | | → | 26.1 | ± | 0.21 | 24.9 | ‑ | 27.5 | 30.5 | ± | 0.38 | 27.1 | - | 34.0 | 31.0 | ± | 0.50 | 28.6 | ‑ | 34.1 | 44.2 | ± | 0.31 | 42.0 | ‑ | 46.3 |
|  | |  | 0.10 | ± | 0.003 | 0.09 | ‑ | 0.12 | 0.18 | ± | 0.007 | 0.13 | - | 0.24 | 0.22 | ± | 0.015 | 0.12 | ‑ | 0.30 | 0.57 | ± | 0.105 | 0.51 | ‑ | 0.63 |
| N1g | | → | 19.3 | ± | 0.30 | 17.8 | ‑ | 20.6 | 21.2 | ± | 0.30 | 19.5 | - | 24.2 | 23.8 | ± | 0.34 | 21.1 | ‑ | 26.2 | 32.3 | ± | 0.28 | 30.1 | ‑ | 34.5 |
|  | |  | 0.05 | ± | 0.002 | 0.04 | ‑ | 0.07 | 0.07 | ± | 0.004 | 0.05 | - | 0.11 | 0.07 | ± | 0.006 | 0.05 | ‑ | 0.12 | 0.25 | ± | 0.009 | 0.20 | ‑ | 0.30 |
| N2 | | → | 19.3 | ± | 0.32 | 17.1 | ‑ | 21.4 | 22.4 | ± | 0.47 | 19.8 | - | 23.7 | 25.1 | ± | 0.71 | 21.3 | ‑ | 29.4 | 34.8 | ± | 0.45 | 32.9 | ‑ | 37.8 |
|  | |  | 0.06 | ± | 0.003 | 0.04 | ‑ | 0.07 | 0.08 | ± | 0.002 | 0.06 | - | 0.17 | 0.11 | ± | 0.013 | 0.06 | ‑ | 0.19 | 0.31 | ± | 0.121 | 0.26 | ‑ | 0.36 |
| N3 | | → | 19.1 | ± | 0.28 | 17.4 | ‑ | 20.4 | 22.4 | ± | 0.39 | 20.3 | - | 24.3 | 27.0 | ± | 0.49 | 25.3 | ‑ | 29.8 | 36.9 | ± | 0.35 | 34.8 | ‑ | 41.0 |
|  | |  | 0.05 | ± | 0.001 | 0.04 | ‑ | 0.07 | 0.08 | ± | 0.002 | 0.05 | - | 0.11 | 0.14 | ± | 0.010 | 0.07 | ‑ | 0.22 | 0.32 | ± | 0.112 | 0.27 | ‑ | 0.37 |
|  | Standard temperature conditions | | | | | | | | | | | | | | | | | | | |  |  |  |  |  |  |
| DV | | → | 20.0 | ± | 0.35 | 17.8 | ‑ | 22.1 | 22.4 | ± | 0.40 | 18.9 | - | 25.3 | 25.8 | ± | 0.55 | 22.2 | ‑ | 29.7 |  |  |  |  |  |  |
|  | |  | 0.05 | ± | 0.002 | 0.04 | ‑ | 0.06 | 0.08 | ± | 0.005 | 0.06 | - | 0.11 | 0.11 | ± | 0.010 | 0.08 | ‑ | 0.19 |  |  |  |  |  |  |
| W | | → | 22.1 | ± | 0.27 | 20.5 | ‑ | 23.9 | 27.8 | ± | 0.37 | 25.0 | - | 30.2 | 29.9 | ± | 0.35 | 28.2 | ‑ | 31.6 |  |  |  |  |  |  |
|  | |  | 0.08 | ± | 0.003 | 0.07 | ‑ | 0.10 | 0.11 | ± | 0.004 | 0.08 | - | 0.15 | 0.16 | ± | 0.009 | 0.12 | ‑ | 0.20 |  |  |  |  |  |  |
| L | | → | 21.3 | ± | 0.23 | 20.6 | ‑ | 21.8 | 26.9 | ± | 0.29 | 24.4 | - | 30.1 | 29.2 | ± | 0.32 | 26.5 | ‑ | 32.0 |  |  |  |  |  |  |
|  | |  | 0.10 | ± | 0.003 | 0.08 | ‑ | 0.12 | 0.13 | ± | 0.006 | 0.09 | - | 0.15 | 0.17 | ± | 0.009 | 0.11 | ‑ | 0.25 |  |  |  |  |  |  |
| N1g | | → | 18.5 | ± | 0.17 | 17.8 | ‑ | 19.7 | 25.5 | ± | 0.33 | 23.3 | - | 26.3 | 26.9 | ± | 0.66 | 23.1 | ‑ | 32.4 |  |  |  |  |  |  |
|  | |  | 0.05 | ± | 0.001 | 0.04 | ‑ | 0.05 | 0.07 | ± | 0.003 | 0.05 | - | 0.10 | 0.12 | ± | 0.011 | 0.07 | ‑ | 0.22 |  |  |  |  |  |  |
| N2 | | → | 18.2 | ± | 0.21 | 17.3 | ‑ | 19.3 | 25.3 | ± | 0.26 | 23.3 | - | 26.2 | 26.4 | ± | 0.46 | 22.4 | ‑ | 28.5 |  |  |  |  |  |  |
|  | |  | 0.05 | ± | 0.001 | 0.04 | ‑ | 0.05 | 0.08 | ± | 0.002 | 0.05 | - | 0.10 | 0.11 | ± | 0.009 | 0.05 | ‑ | 0.16 |  |  |  |  |  |  |
| N3 | | → | 19.1 | ± | 0.17 | 18.0 | ‑ | 20.1 | 25.2 | ± | 0.28 | 23.5 | - | 26.0 | 26.1 | ± | 0.24 | 23.3 | ‑ | 28.5 |  |  |  |  |  |  |
|  | |  | 0.05 | ± | 0.001 | 0.05 | ‑ | 0.06 | 0.08 | ± | 0.002 | 0.05 | - | 0.09 | 0.10 | ± | 0.008 | 0.05 | ‑ | 0.15 |  |  |  |  |  |  |

Note. The live egg diameter is shown instead of FL.
